# A trap mutant reveals the physiological client spectrum of TRC40

**DOI:** 10.1242/jcs.230094

**Published:** 2019-07-01

**Authors:** Javier Coy-Vergara, Jhon Rivera-Monroy, Henning Urlaub, Christof Lenz, Blanche Schwappach

**Affiliations:** 1 Department of Molecular Biology, University Medical Center Göttingen, Göttingen 37073, Germany; 2 Bioanalytical Mass Spectrometry Group, Max Planck Institute for Biophysical Chemistry, Göttingen 37077, Germany; 3 Bioanalytics Group, Institute of Clinical Chemistry, University Medical Center Göttingen, Göttingen 37077, Germany; 4 Max Planck Institute for Biophysical Chemistry, Göttingen 37077, Germany

**Keywords:** TRC40, Chaperone, Client spectrum, Endoplasmic reticulum, Membrane targeting, Tail-anchored protein

## Abstract

The transmembrane recognition complex (TRC) pathway targets tail-anchored (TA) proteins to the membrane of the endoplasmic reticulum (ER). While many TA proteins are known to be able to use this pathway, it is essential for the targeting of only a few. Here, we uncover a large number of TA proteins that engage with TRC40 when other targeting machineries are fully operational. We use a dominant-negative ATPase-impaired mutant of TRC40 in which aspartate 74 was replaced by a glutamate residue to trap TA proteins in the cytoplasm. Manipulation of the hydrophobic TA-binding groove in TRC40 (also known as ASNA1) reduces interaction with most, but not all, substrates suggesting that co-purification may also reflect interactions unrelated to precursor protein targeting. We confirm known TRC40 substrates and identify many additional TA proteins interacting with TRC40. By using the trap approach in combination with quantitative mass spectrometry, we show that Golgi-resident TA proteins such as the golgins golgin-84, CASP and giantin as well as the vesicle-associated membrane-protein-associated proteins VAPA and VAPB interact with TRC40. Thus, our results provide new avenues to assess the essential role of TRC40 in metazoan organisms.

This article has an associated First Person interview with the first author of the paper.

## Abstract (precis)

**Summary:** A strategy to decipher which tail-anchored proteins do (as opposed to can or must) use the TRC pathway in intact cells generates a comprehensive list of human TRC40 clients.

## INTRODUCTION

The presence of membrane proteins customizes biological membranes to suit their respective functions beyond the formation of a permeability barrier. Membrane proteins can enable transmembrane transport, modify the lipid environment or mediate membrane contacts. This is true for the plasma membrane as well as for the many intracellular membranes enclosing subcellular compartments. The integration of a protein into a membrane-forming lipid bilayer is a feat of nature since protein biogenesis occurs in the aqueous phase. Investigation of the cellular machineries that enable the integration of proteins into membranes is as much a classical area as a current focus of molecular cell biology.

Reductionist approaches afford tremendous insights into the fundamentals of membrane integration of proteins. In fact, *in vitro* reconstitution (Kutay et al., 1995; Stefanovic and Hegde, 2007) and the theoretical, physicochemical analysis of transmembrane segments (TMS) (von Heijne, 2007) have enabled the field to formulate detailed hypotheses about rules that govern membrane integration of proteins. In parallel, systematic strategies have uncovered several different protein complexes and pathways involved in membrane targeting at large (Aviram et al., 2016; Nguyen et al., 2018; Schuldiner et al., 2008; Shurtleff et al., 2018). These discoveries raise questions with regard to redundancy (which may be in place to achieve robustness), tissue specificity (which may optimize the physiological function of specialized cell types) and the hierarchy of different membrane protein targeting pathways. Investigation of different cell types by physiologically minded *in vivo* approaches has revealed that some substrates are particularly sensitive to the loss of a specific pathway (Casson et al., 2017; Norlin et al., 2016; Rivera-Monroy et al., 2016; Vogl et al., 2016).

An interesting example is provided by the topologically defined class of tail-anchored (TA) proteins, which comprise one hydrophobic TMS at the C-terminus that anchors the protein in the membrane. The cytoplasmic N-terminus endows such proteins with their diverse functions, for example, in vesicular traffic (most SNARE proteins are TA proteins) whereas the luminal C-terminus does not exceed 40 amino acids (Borgese et al., 2003; Kutay et al., 1993). TA proteins occur on all membranes facing the cytoplasm. The TA proteins found in the contiguous secretory pathway, plasma membrane and endo-lysosomal system are thought to be integrated into the membrane of the endoplasmic reticulum (ER) before they reach their respective locales by vesicular traffic (Borgese et al., 2007; Jäntti et al., 1994; Kutay et al., 1995; Linstedt et al., 1995; Pedrazzini et al., 1996).

The yeast GET or mammalian transmembrane recognition complex (TRC) pathway has been identified as a highly conserved cellular pathway capable of targeting TA proteins to the ER (Favaloro et al., 2008; Mariappan et al., 2010; Schuldiner et al., 2008; Stefanovic and Hegde, 2007). A plethora of complementary data from systematic genetic analyses in yeast (Jonikas et al., 2009; Schuldiner et al., 2008), *in vitro* biochemical dissection (Bozkurt et al., 2009; Favaloro et al., 2008, 2010; Stefanovic and Hegde, 2007) and numerous crystal structures (Bozkurt et al., 2009; Gristick et al., 2014; Hu et al., 2009; Kubota et al., 2012; Mateja et al., 2009, 2015; Simpson et al., 2010; Stefer et al., 2011; Yamagata et al., 2010) have helped generate the current model of TRC pathway function. However, it has become equally clear that *in vivo* the TA proteins destined for the ER have several additional targeting options, such as targeting being mediated by the signal recognition particle (SRP) system (Abell et al., 2004, 2007; Casson et al., 2017), the SRP-independent targeting (SND) components (Aviram et al., 2016; Haßdenteufel et al., 2017) and the ER membrane complex (EMC) (Guna et al., 2018; Shurtleff et al., 2018). Hence, it is necessary to carefully distinguish which TA proteins can use the TRC pathway from the precursors for which it is essential. While the existing tools such as *in vitro* assays and knockout cells or organisms have begun to provide answers to these questions (Casson et al., 2017; Figueiredo Costa et al., 2018; Guna et al., 2018; Haßdenteufel et al., 2017; Lin et al., 2016; Norlin et al., 2016, 2018; Pfaff et al., 2016; Rivera-Monroy et al., 2016; Vogl et al., 2016), an approach is still lacking that would reveal which TA proteins do in fact use the TRC pathway when all targeting options are operational (i.e. without the compensatory effects often provoked by knockout strategies).

Here, we present a novel strategy to tackle this problem. Based on the ATPase cycle of TRC40, we designed and characterized a dominant negative ‘trap’ mutant of TRC40, which leads to the accumulation of TRC40-interacting precursor TA proteins in the cytoplasm. This approach helped us to confirm known substrates of the pathway, to distinguish precursors that can but, importantly, do not have, to use TRC40-dependent membrane integration, and to test a large spectrum of endogenously expressed TA proteins for their capacity to interact with TRC40 in the intact cellular environment. In addition, unbiased analysis of the interactome of the TRC40 trap mutant, by label-free quantitative mass spectrometry, identified more than ten TA proteins interacting with TRC40. Among these newly identified and subsequently verified TA proteins are golgins such as golgin-84 (also known as GOLGA5), CASP (encoded by *CUX1*; isoform 4) and giantin (also known as GOLGB1). Golgins are central to the organization and functionality of the Golgi (Gillingham and Munro, 2016). Given the crucial role of giantin in the posttranslational modification of secretory proteins through mediating glycosylation (Lan et al., 2016; McGee et al., 2017; Stevenson et al., 2017), our finding may help to explain the poorly understood essentiality of the TRC pathway in mammals. At the same time, our data suggest that the observed co-purification of TA proteins with TRC40 may partially reflect a molecular interaction unrelated to precursor protein targeting. Hence, our analysis motivates future focus on the chaperone function of proteins homologous to TRC40 and its yeast counterpart Get3 (Farkas et al., 2019).

## RESULTS

### Steady-state levels of TA proteins upon combined WRB and TRC40 depletion report on TRC pathway substrates with limited fidelity

Cellular quality control promotes the degradation of membrane protein precursors that mislocalize because their preferred targeting pathway is not operational (reviewed in Guna and Hegde, 2018). This consequence of mistargeting has been exploited to interpret the reduced steady-state levels of a TA protein upon knockout or siRNA-mediated knockdown of TRC pathway components as evidence for the targeting of the respective substrate via TRC40 (Casson et al., 2017; Haßdenteufel et al., 2017; Norlin et al., 2016; Rivera-Monroy et al., 2016). In combination with *in vitro* insertion assays, which answer the question of whether a given substrate can target to the membrane of the ER via a TRC40, and WRB and CAML-dependent route (CAML is also known as CAMLG), the results of this approach have been useful to identify true substrates of the pathway, for which it is obligatory, such as syntaxins Stx5 and Stx6 as well as emerin (EMD) (Norlin et al., 2016; Pfaff et al., 2016; Rivera-Monroy et al., 2016).

**Fig. 1.**
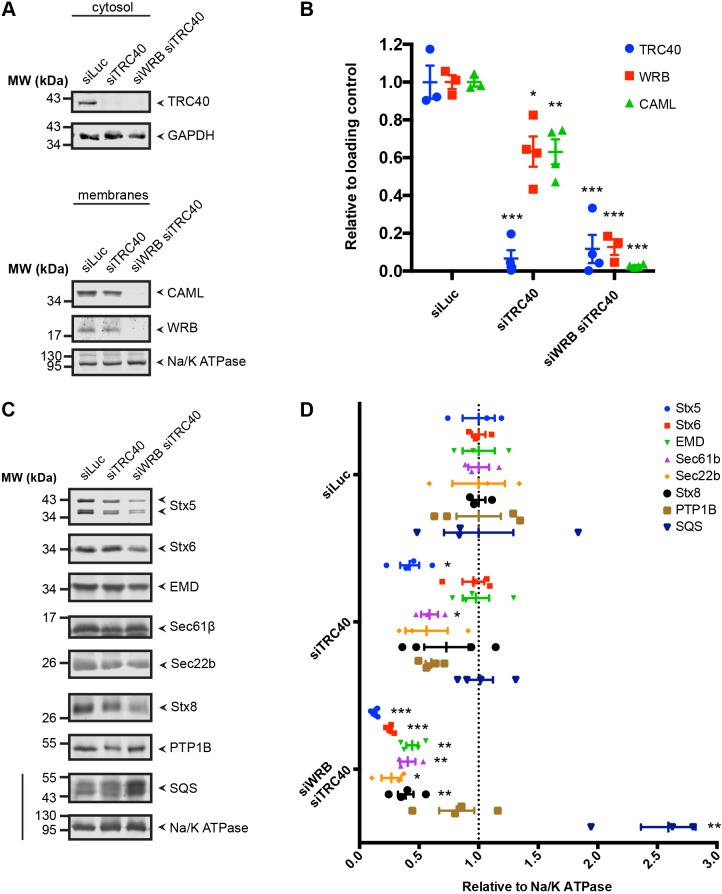
**Silencing *WRB* and *TRC40* affects the steady-state levels of many TA proteins.** (A) Western blots demonstrating the downregulation of *TRC40* and *WRB* upon transfection with previously characterized siRNAs (Pfaff et al., 2016; Rivera-Monroy et al., 2016; Yamamoto and Sakisaka, 2012) in membrane and cytosol fractions. (B) Quantifications of western blots shown in A (*n*≥3, mean±s.e.m. shown). (C) Western blots of the membrane fraction to detect steady-state levels of different TA proteins after silencing *TRC40* or *TRC40* and *WRB*. The line indicates that the two panels are derived from the same lanes of the same gel. All panels have a pertinent loading control used for the quantification but not shown here. (D) Quantifications of western blots shown in C (*n*≥3, mean±s.e.m. shown). *P<0.05; **P<0.01; ***P<0.001 relative to siLuc (two-tailed *t*-test).

By using the above strategy in HeLa cells, we assessed the steady-state levels of TA proteins that were implied as TRC pathway-dependent substrates through *in vitro* assays but not definitively and systematically tested as substrates in intact cells (Fig. 1). Substantial knockdown of *TRC40* and *WRB* was achieved (Fig. 1A,B) and the combined transfection with siRNAs directed against TRC40 and WRB resulted in a robust reduction of the TRC40 ATPase and both the TRC40 receptor proteins WRB and CAML. In effect, this manipulation leads to a knockout of the membrane-targeting phase of the TRC40 cycle, in line with results obtained from the analysis of a *Wrb*-knockout mouse (Rivera-Monroy et al., 2016). As a positive control, we recapitulated TA protein substrates known to depend on the TRC pathway *in vivo* such as Stx5, Stx6 and EMD, which showed reduced steady-state levels and also aberrant subcellular localization when the pathway is impaired (Norlin et al., 2016; Pfaff et al., 2016; Rivera-Monroy et al., 2016). Indeed, the steady-state levels of all three substrates in the membrane fraction were strongly reduced upon downregulation of *TRC40* and *WRB* (Fig. 1C,D).

Next, we tested three TA proteins, Sec61β, Sec22b and Stx8 (Fig. 1C,D), for which good *in vitro* evidence exists that they – or their highly conserved yeast orthologs – require the mammalian TRC or yeast GET pathway (Jonikas et al., 2009; Rivera-Monroy et al., 2016; Schuldiner et al., 2008; Stefanovic and Hegde, 2007; Stefer et al., 2011; Yamagata et al., 2010). However, the *in vivo* reliance of these proteins on the pathway has been reported to be variable or they turned out to be independent of TRC40 and WRB (Casson et al., 2017; Norlin et al., 2016; Rivera-Monroy et al., 2016). In HeLa cells, Sec61β, Sec22b and Stx8 displayed reduced steady-state levels in the membrane fraction upon the combined downregulation of *TRC40* and *WRB* (Fig. 1C,D). Interestingly, previous work reported correct localization of Sec61β in *TRC40*- or *WRB*-knockout cells or mouse tissue (Casson et al., 2017; Norlin et al., 2016; Rivera-Monroy et al., 2016) indicating that assays of subcellular localization may report less sensitively on an involvement of the TRC pathway than assessments of steady-state protein levels.

As a negative control for the assay, we included the TA protein PTP1B (also known as PTPN1), which is able to insert spontaneously into liposomes (Brambillasca et al., 2006), and squalene synthase (SQS, also known as FDFT1), where targeting has recently shown to be independent of TRC40 (Guna et al., 2018; Shurtleff et al., 2018). For PTP1B no significant changes were observed upon silencing *TRC40* or both *WRB* and *TRC40*, although the scatter of the observed signal does not exclude subtle effects (Fig. 1C,D). SQS steady-state levels increased upon combined knockdown of *TRC40* and *WRB*. The molecular mechanisms behind the steady-state levels of a protein may be complex. On the one hand, compensatory changes in other targeting pathways (Haßdenteufel et al., 2017) may hide TRC pathway dependence. On the other hand, effects downstream of *bona fide* targeting substrates, such as Stx5, may suggest targeting dependence of a complex partner whose stability is affected by the targeting client. Indeed, Sec22b is a SNARE partner protein of Stx5 (Hay et al., 1997) and its steady-state levels may be indirectly affected by the combined downregulation of *WRB* and *TRC40*. For PTP1B and SQS, our results are consistent with the notion that they do not require the TRC pathway for correct targeting.

**Fig. 2.**
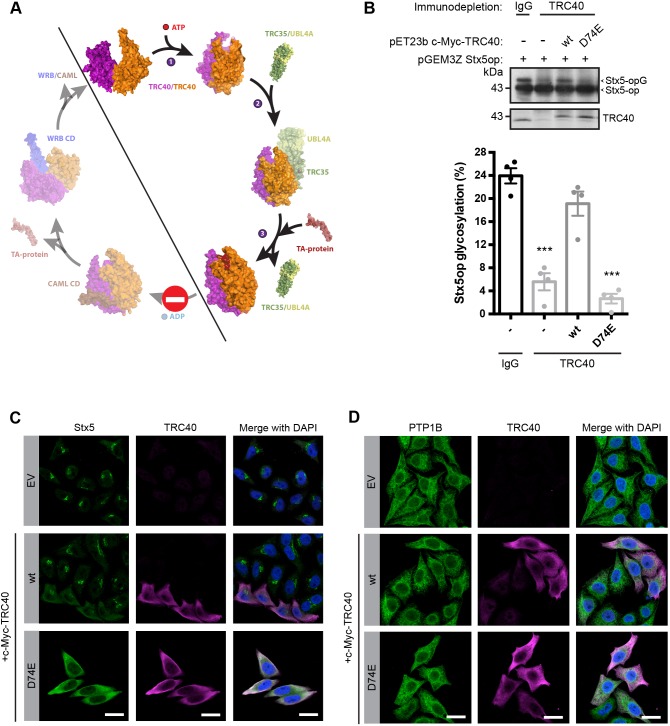
**Overexpression of TRC40_D74E_ leads to the mislocalization of Stx5.** (A) Model of the TRC40 ATPase cycle based on structures representing different intermediates in the ATPase cycle of yeast Get3. The Protein Data Bank (PDB) IDs used for this scheme are as follows: 3H84, 2WOJ, 4PWX, 2LPF, 4XTR, 3ZS9 and 3SJB. CD stands for cytosolic domain. (B) Stx5 insertion into canine microsomes was detected by assessing the glycosylation of the C-terminal opsin tag. Opsin-tagged Stx5 was *in vitro* translated in mock-depleted or TRC40-immunodepleted rabbit reticulocyte lysate. In these lysates, c-Myc–TRC40 variants were first translated *in vitro* followed by the *in vitro* translation of opsin-tagged Stx5. Western blotting was performed detecting the indicated proteins. The graph shows a quantification of Stx5 glycosylation as percentage of total (*n*=4, mean±s.e.m. shown). ***P<0.001 (two-tailed *t*-test). (C,D) Immunofluorescence analysis of the subcellular localization of TA proteins in HeLa cells overexpressing c-Myc–TRC40 variants. Transfection with the empty vector (EV) was used as negative control. Cells were fixed and stained for c-Myc–TRC40 (magenta, C,D), Stx5 (green, C), PTP1B (green, D) (representative of *n*≥4). Scale bars: 20 µm.

To avoid conclusions based on steady-state TA protein levels alone, we developed a complementary approach to tackle the question of whether a substrate displaying *in vitro* dependence and *in vivo* or *in cellulo* independence of TRC40 does use the pathway when it is available. To more directly probe the propensity of a TA protein precursor to be targeted by the TRC pathway, we were inspired by approaches from the protease, protein disulfide isomerase, AAA ATPase and GTPase fields that have used dominant-negative forms of the respective enzyme to trap transiently interacting substrates (Chen et al., 2014; Flynn et al., 2003; Jessop et al., 2009; Klebe et al., 1995). Based on the current model of the TRC40 ATPase cycle and the associated conformations of the protein (Fig. 2A), we set out to address the flux through the TRC pathway by a substrate-trapping approach.

### Expression of ATPase-impaired TRC40_D74E_ alters the subcellular localization of TA proteins

TRC40 and its yeast ortholog Get3 are members of the SIMIBI family of NTPases, which also includes the SRP (Bange and Sinning, 2013). In contrast to the GTPase activity of SRP, Get3 is an ATPase that undergoes large conformational changes in response to ATP binding and hydrolysis (Fig. 2A). Hence, several residues involved in ATP binding or hydrolysis have been targeted to experimentally manipulate Get3. Several studies have used the Get3_G30R_ mutant (Johnson et al., 2013; Schuldiner et al., 2005; Shen et al., 2003; Suloway et al., 2009; Wang et al., 2011b), which targets a residue in the P-loop or Walker A motif and is predicted to be deficient in nucleotide binding (Saraste et al., 1990). Another extensively used mutant is Get3_D57N_, which targets a residue in the conserved Switch I ATPase domain. This mutation impairs the ATPase activity of Get3 (Chio et al., 2017; Mateja et al., 2009; Stefer et al., 2011; Wang et al., 2011a) rather than abolishing nucleotide binding. Based on the effects of changing the orthologous D45 in the Switch I domain of the bacterial homolog ArsA (Zhou and Rosen, 1999), we previously created a Get3_D57E_ mutant, demonstrated its strongly impaired ATPase activity, and showed its inability to substitute for wild-type Get3 in yeast, although the protein was well-expressed (Powis et al., 2013). The current model of the Get3 ATPase cycle and, by extension TRC40 (Fig. 2A), predicts that a form of the protein that can bind but not hydrolyze ATP may capture but not release TA protein substrates (Chio et al., 2017; Mariappan et al., 2011; Stefer et al., 2011). Our previous results with yeast Get3_D57E_ (Powis et al., 2013), specifically the colocalization of Get3_D57E_–GFP and a TA protein substrate in intracellular foci, are consistent with the idea that TA protein precursors may be trapped by this mutant in cells. To make use of this tool in the mammalian system, where many more TA proteins exist (Kalbfleisch et al., 2007), we constructed and characterized the homologous TRC40_D74E_ mutant.

Originally, small TA proteins such as Sec61β (Stefanovic and Hegde, 2007) or Ramp4 (Favaloro et al., 2008) were used as model substrates to identify the mammalian TRC pathway by using an *in vitro* membrane insertion assay and protein crosslinking. However, the SNARE protein Stx5 is the best-characterized TRC40 substrate that critically depends on the presence of the pathway *in vivo* (Casson et al., 2017; Norlin et al., 2016, 2018; Rivera-Monroy et al., 2016). Likewise, yeast syntaxin 5 (Sed5) requires Get3 for proper localization and targeting (Jonikas et al., 2009; Powis et al., 2013; Schuldiner et al., 2008; Voth et al., 2014). Therefore, we used a C-terminally opsin-tagged variant Stx5 as a substrate to assess the impact of TRC40_D74E_ on TA protein insertion in an *in vitro* insertion assay (Fig. 2B). The opsin tag is an N-glycosylation tag that corresponds to 13 amino acids of bovine opsin. It becomes glycosylated on the luminal side once the protein is inserted into the ER. Thereby it enables monitoring of the ER insertion of the protein (Abell et al., 2007; Borgese et al., 2001; Favaloro et al., 2010; Honsho et al., 1998; Kutay et al., 1995; Masaki et al., 1996; Pedrazzini et al., 2000; Schuldiner et al., 2008; Stefanovic and Hegde, 2007). Reticulocyte lysate was immunodepleted of TRC40, which strongly reduced glycosylation and hence insertion of Stx5 (Fig. 2B). Re-expressing either wild-type TRC40 or TRC40_D74E_ in the immuno-depleted lysate revealed that TRC40_D74E_ was not capable of mediating the insertion of Stx5 (Fig. 2B). Instead, there was in fact a reduction in the small amount of glycosylated and, hence, inserted, Stx5 observed in the immunodepleted sample.

In mammalian cells and in yeast, impairing the TRC/GET pathway leads to a loss of Stx5/Sed5 from the Golgi (Casson et al., 2017; Norlin et al., 2016; Rivera-Monroy et al., 2016; Schuldiner et al., 2008). Therefore, we used the subcellular localization of endogenous Stx5, as determined by indirect immunofluorescence, as a readout to evaluate the effects of TRC40_D74E_ overexpression (Fig. 2C). We transfected HeLa cells with c-Myc-tagged TRC40 constructs and performed double-labeling immunofluorescence staining for Stx5 and c-Myc–TRC40. Upon transfection with the TRC40_D74E_ mutant, Stx5 changed its subcellular localization showing a diffuse, apparently cytoplasmic, staining instead of the Golgi staining that reflects correct membrane targeting and subsequent sorting of Stx5. Overexpression of wild-type TRC40 (TRC40_wt_) showed a partially cytoplasmic staining for Stx5 (Fig. 2C), in line with the idea that the conformation stabilized by TRC40_D74E_ reflects a step of the normal TRC40 cycle that can also be populated more by raising the TRC40 levels. Alternatively, overexpression of TRC40 may create a large pool of the ATPase free of TA protein substrate. This form of TRC40 may titrate out the binding sites on the receptor required for proper Stx5 targeting. By using the TRC40_D74E_-based assay, we revisited the TA protein PTP1B, whose steady-state levels were not significantly affected by silencing the TRC pathway (Fig. 1C,D), and observed no obvious effects on its localization pattern (Fig. 2D).

To corroborate the trapping function of the D74E mutant, we tested another presumably ATPase-deficient mutant, D74N, which was previously reported to constitutively bind TA proteins in the background of Get3 (Mateja et al., 2009, 2015; Stefer et al., 2011). Overexpression of TRC40_D74N_ resulted in the same change of Stx5 subcellular localization (Fig. S1) that we had observed in the presence of TRC40_D74E_ (Fig. 2C). This observation is in line with a recent report showing that TRC40_D74N_ failed to restore proper distribution of Stx5 in dorsal pancreas explants (Norlin et al., 2018). We tested another *bona fide* TRC40 substrate, EMD (Pfaff et al., 2016; Rivera-Monroy et al., 2016), and observed a diffusely distributed population when staining the TRC40_D74E_-transfected cells for endogenous EMD (Fig. S2). Taken together, these results strengthen the notion that overexpression of TRC40 mutants, in which ATP-binding capacity is preserved but ATPase activity is impaired, may divert the TA protein substrate from its subcellular destination, the Golgi membrane in the case of Stx5 or the inner nuclear membrane in the case of EMD.

### Over-expression of TRC40_D74E_ causes cytoplasmic accumulation of TA proteins

**Fig. 3.**
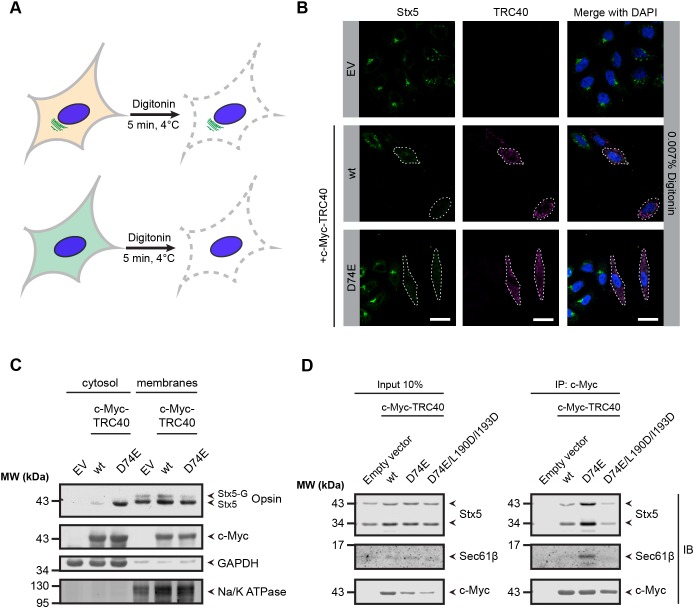
**In the presence of TRC40_D74E_ Stx5 accumulates in the cytoplasm.** (A) Experimental strategy for testing whether Stx5 was washed out in TRC40_D74E_-transfected cells after treatment with digitonin. (B) Cells were semi-permeabilized in the presence of a buffer containing 0.007% digitonin and then fixed. Cells transfected as indicated were subjected to indirect immunofluorescence using antibodies against c-Myc (magenta) and Stx5 (green). Dashed lines outline c-Myc–TRC40-transfected cells. Scale bars: 20 µm. (C) Stx5 accumulated in the cytosol of cells overexpressing TRC40_D74E_. Cell lysates from the Stx5–opsin stable cell line were subjected to fractionation. Cells were transfected with either c-Myc–TRC40 or c-Myc–TRC40_D74E_. Transfection with the empty vector (EV) was used as negative control. Subsequently, the expression of Stx5–opsin was induced with 1 µg/ml of tetracycline (Tet) for 6 h. Western blotting was performed detecting the indicated proteins. (D) Co-immunoprecipitation from cytosol in the absence of detergent shows that TRC40_D74E_ interacts with TA proteins such as Stx5 and Sec61β. Cells were transfected with c-Myc–TRC40 variants using the empty vector (EV) as negative control. Western blotting was performed detecting the indicated proteins. Note that endogenous Stx5 migrates as two different bands due to alternative start codons (Hui et al., 1997). All results representative of *n*=3.

In order to analyze whether the diffuse staining pattern observed for Stx5 and EMD in TRC40_D74E_-transfected cells (Fig. 2C; Fig. S2) reflected localization to the cytoplasm, we semi-permeabilized cells with digitonin before immunofluorescence staining (Fig. 3A). Digitonin preferentially permeabilizes the plasma membrane leaving the rest of the cell membranes intact (Plutner et al., 1992; Wilson et al., 1995). In untransfected cells, Stx5 was observed in its typical Golgi staining pattern (Fig. S3) upon digitonin semi-permeabilization but – as expected for cytoplasmic components – the diffuse Stx5 signal observed was washed out from TRC40_D74E_-transfected cells and partially from TRC40_wt_-transfected cells (Fig. 3B). This experiment indicates that Stx5 accumulates in the cytoplasm upon overexpression of TRC40_D74E_.

To further corroborate this conclusion, we developed a stable cell line (Flp-In T-REx-293) with an opsin-tagged Stx5 at the C-terminus under the control of a tetracycline-inducible promoter. In order to biochemically investigate whether Stx5 is accumulated in the cytosol, as indicated by its cytoplasmic localization observed through immunofluorescence, we transfected the Stx5–opsin (Stx5–op) stable cell line with c-Myc-tagged TRC40 constructs before induction of the opsin-tagged substrate to create a pulse of Stx5 precursors. Next, we performed subcellular fractionation and analyzed the different fractions by western blotting. Stx5-op steady-state levels were higher in the cytosolic fraction of the cells transfected with TRC40_D74E_ than in those transfected with the empty vector (Fig. 3C), corroborating the observations obtained by microscopy. There was one strong band reflecting Stx5–op in the cytosolic fraction as opposed to the membrane fraction where we observed two bands (Fig. 3C). The single band observed in the cytosol is consistent with the interpretation that an unglycosylated biogenetic precursor was trapped. Furthermore, analysis of the membrane fraction suggested less Stx5–op was glycosylated and hence inserted into the membrane in the presence of overexpressed TRC40_D74E_ as compared to when TRC40_wt_ was overexpressed. These findings raise the possibility that TRC40_D74E_ interacts with Stx5 in the cytoplasm and acts as a trap mutant, impeding its handover to the TRC receptor to enable membrane insertion.

Therefore, we tested whether TRC40_D74E_ and endogenous Stx5 physically interact. We transfected HEK293 cells with c-Myc–TRC40 constructs and immunoprecipitated c-Myc–TRC40 from the cytosol in the absence of detergent, which was then probed for co-precipitating endogenous Stx5 (Fig. 3D). We also probed for Sec61β to test a candidate substrate that certainly can target via the TRC pathway, as demonstrated by previous *in vitro* assays (Favaloro et al., 2008, 2010; Stefanovic and Hegde, 2007), and is affected in its steady-state levels upon TRC pathway silencing (Fig. 1C,D), but does not depend on the TRC pathway for proper subcellular localization (Casson et al., 2017; Rivera-Monroy et al., 2016). To corroborate our interpretation that TRC40_D74E_ acts as a substrate trap, we introduced two additional mutations into the plasmid encoding the protein. These have been shown to reduce the affinity of the TA-binding groove – formed by the TRC40 dimer – for the TMS of the TA protein (Mateja et al., 2009; Shao et al., 2017). The resulting c-Myc–TRC40_D74E/L190D/I193D_ poorly co-precipitated Stx5 and Sec61β (Fig. 3D). Consistent with the notion that the diffuse Stx5 staining pattern in TRC40_D74E_-transfected cells reflects a cytoplasmic population in complex with TRC40, the Golgi localization of Stx5 was partially rescued in the presence of the triple mutant TRC40_D74E/L190D/I193D_ (Fig. S3). The effect of restoring the proper subcellular localization by combining the D74E mutation with the additional two mutations, which reduce the binding affinity of the TRC40 to the TMSs of TA proteins, was very clear for EMD (Fig. S2). Thus, the cytosolic interaction, and hence the diffuse staining pattern observed for substrates in the presence of TRC40_D74E_, correlated with an intact TA-binding groove, which is consistent with our rationale to stabilize the TA-protein-interacting form of TRC40. It should, however, be noted that much less is currently known about the molecular details of how Get3-like chaperones interact with their clients (Farkas et al., 2019; Voth et al., 2014). Hence, we cannot exclude that some of the effects of overexpressing TRC40_D74E_ may not directly relate to membrane targeting of TA protein precursors during biogenesis.

### Characterization of TA protein interactome of TRC40_D74E_

Based on our characterization of TRC40_D74E_ as a substrate trapping mutant, we applied this tool in two independent, complementary strategies to map the TRC40 client spectrum. On the one hand, we used a large panel of antibodies (Table S1) to test the presence of TA proteins in the c-Myc–TRC40_D74E_ immunoprecipitates (Fig. S4). As with the silencing approach (Fig. 1; Fig. S7), this approach is limited by the quality of the antibodies available. On the other hand, we conducted an unbiased mass spectrometry-based experiment and transfected HEK293 cells with c-Myc–TRC40_D74E_ or the corresponding triple mutant TRC40_D74E/L190D/I193D_, which reduces the affinity of the TMS-binding groove for TA protein substrates (Fig. 3D). After subcellular fractionation and anti-c-Myc immunoprecipitation from the cytosol in the absence of detergent, the two eluates were analyzed by unbiased label-free mass spectrometry. Qualitatively, 2329 proteins out of 3135 detected proteins were identified in both eluates, including 18 TA proteins (Table S2). Since 95 TA proteins were detected in whole-cell lysates of HEK293 cells (Geiger et al., 2012), we identified 20% of all robustly expressed TA proteins in the cytosol of the transfected HEK293 cells. The two eluates were then quantitatively compared by sequential window acquisition of all theoretical fragment ion spectra (SWATH) (Lambert et al., 2013) mass spectrometry (Table S3 lists the peptides used for quantification). We corroborated the results by performing western blot analysis of individual c-Myc–TRC40 proteins and determining the amounts of co-immunoprecipitated Stx5 (Figs S4 and S5). Both approaches unequivocally confirmed the results obtained in Figs 2 and 3 (i.e. the trapping of Stx5 by TRC40_D74E_), and hence the suitability of the approach to identify TA proteins that do interact with TRC40.

**Fig. 4.**
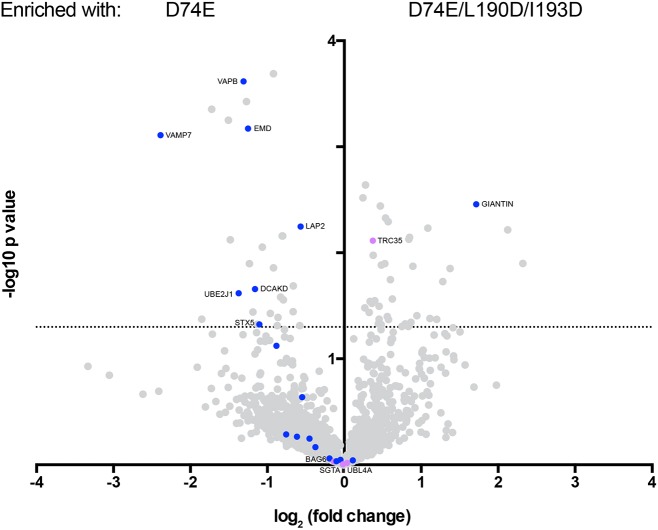
**Unbiased SWATH mass spectrometry analysis reveals additional TA substrates.** TRC40_D74E_ TA protein enrichment compared to the mutant manipulating the TA-binding groove TRC40_D74E/L190D__/I193D_. Volcano plot for c-Myc–TRC40 SWATH-based interactome (results at mean for *n*=3). TRC pathway components are labeled in magenta and TA proteins in blue. The dotted line indicates statistical significance (*P*<0.05; by two-tailed *t*-test).

The results of the SWATH mass spectrometry approach are depicted in a volcano plot of the enrichment, with the single or triple TRC40 mutant plotted against the statistical significance of the result (Fig. 4). All components of the mammalian pre-targeting complex [i.e. SGTA, BAG6, UBL4A and TRC35 (magenta dots in Fig. 4)] were detected in the eluates. Except for a possible modest enrichment of TRC35 with c-Myc–TRC40_D74E/L190D/I193D_, these proteins showed no preference for either form of TRC40. With the exception of the golgin giantin, all TA proteins (blue dots in Fig. 4, Table S2) were either present in equal amounts or enriched with c-Myc–TRC40_D74E_. The positive control protein Stx5 was enriched ∼2-fold and right at the significance limit (*P*<0.05). Focusing on the six TA proteins that were enriched similarly or more strongly than Stx5 (Fig. 4, Table S2), namely, UBE2J1, LAP2B, VAMP7, EMD and VAPB (DCAKD was not studied further due to the unavailability of a specific antibody), we found all five to specifically co-immunoprecipitate with c-Myc–TRC40_D74E_ (Table 1; Fig. S4A). As expected for EMD, based on the literature (Pfaff et al., 2016) and the results presented here (Fig. 1, Fig. S2), all five TA proteins were confirmed as TRC40 interactors contacting the TA-binding groove (Fig. S4A).

**Table 1.**
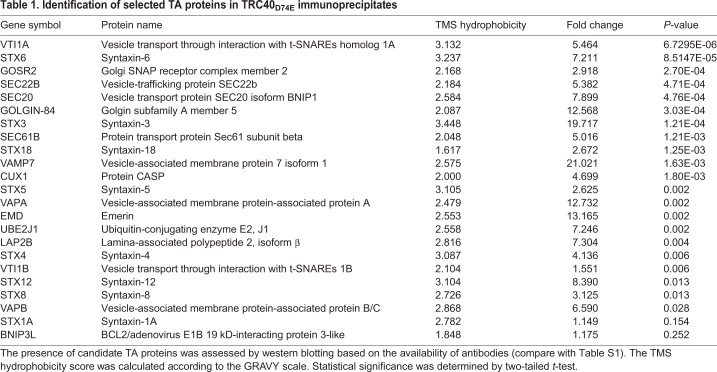
**Identification of selected TA proteins in TRC40_D74E_ immunoprecipitates**

The candidate-based approach using antibodies affords a much higher sensitivity for detecting TA proteins in the TRC40 immunoprecipitates. By using this strategy, we were able to substantially extend the number of tested TA proteins. In addition to Stx5, Sec61β and the five TA proteins tested after identification by the SWATH mass spectrometry approach (EMD, VAPB, VAMP7, LAP2B and UBE2J1), we were able to obtain meaningful data regarding an additional 22 TA proteins (Table 1; Fig. S4B,C). Importantly, this approach also revealed seven TA proteins that were not trapped by TRC40_D74E_ although they were robustly detected in whole-cell lysates (Fig. S4C). The total list of 30 probed TA proteins is a valuable resource when corroborating or refuting rules that determine the TRC40 dependence of a client.

We confirmed the capacity to create a diffuse, presumably cytoplasmic, pool due to trapping by TRC40_D74E_ for two additional syntaxins (Fig. S6). Stx6 is a SNARE protein involved in Golgi trafficking (Bock et al., 1996; Cheng et al., 2010) and has previously been implicated as a *bona fide* TRC40 substrate, with manipulation of the pathway *in vivo* affecting its steady-state levels and localization (Norlin et al., 2016; Rivera-Monroy et al., 2016). Stx8 is a SNARE protein localized at endosomes (Kasai et al., 2008; Prekeris et al., 1999; Subramaniam et al., 2000). Interestingly, its steady-state levels were not affected in the liver or the ventricle of the *Wrb*-knockout mouse and its subcellular localization indicated robust membrane targeting (Rivera-Monroy et al., 2016). Although the two syntaxins reacted differently to the loss of the TRC pathway in these mouse tissues, they were both enriched in the TRC40_D74E_ immunoprecipitation (Fig. S4B; Table 1). In line with this observation, we observed a partially diffuse staining pattern for endogenous Stx6 (Fig. S6A) and Stx8 (Fig. S6B) upon overexpression of the TRC40 trap. Many of the antibodies used for western blotting were not suitable for indirect immunofluorescence, which is why we limited ourselves to confirming the appearance of a presumably cytoplasmic pool for selected TA proteins (Stx5, Stx6, Stx8 and EMD). In all cases, physical interaction with TRC40_D74E_ was predictive of the aberrant subcellular localization that is consistent with substrate trapping in the cytoplasm.

**Fig. 5.**
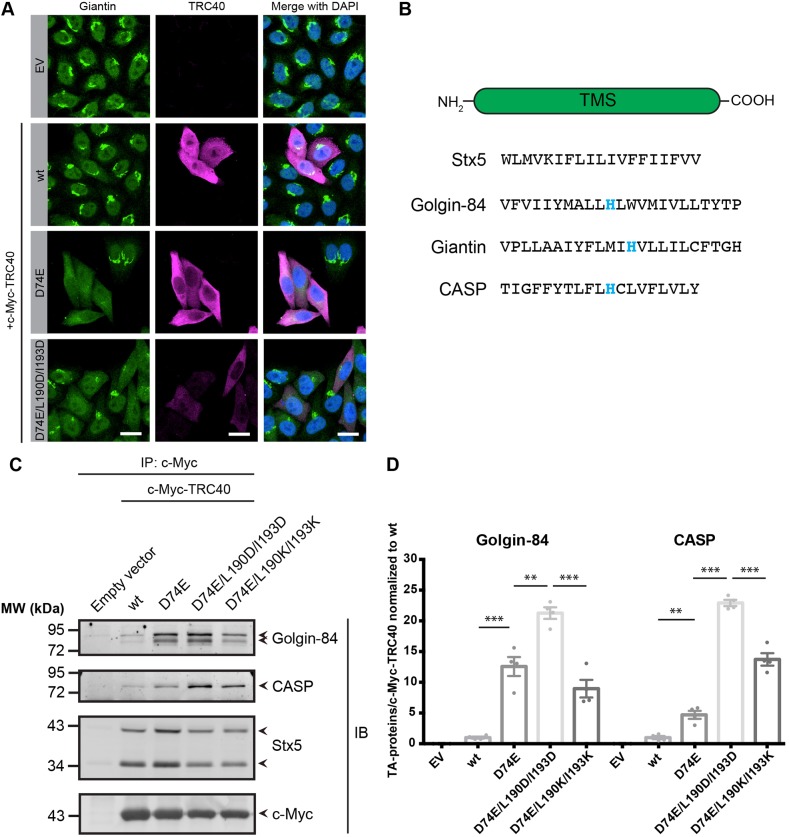
**TA golgins are trapped by TRC40_D74E_.** (A) HeLa cells were transfected to express c-Myc–TRC40 constructs and later processed for indirect immunofluorescence using antibodies against c-Myc (magenta) and giantin (green) (representative of *n*=3). Transfection with the empty vector (EV) was used as negative control. Scale bars: 20 µm. (B) Comparison of TMS of the TA golgins. (C) Co-immunoprecipitation from cytosol in the absence of detergent shows that TRC40_D74E_ interacts with TA proteins such as golgin-84 and CASP. Cells were transfected with c-Myc–TRC40 variants, using the empty vector (EV) as negative control. Western blotting was performed detecting the indicated proteins. (D) Quantifications of western blots shown in C (*n*=4, mean±s.e.m. shown). **P<0.01; ***P<0.001 (two-tailed *t*-test).

One TA protein – the coiled-coil forming golgin giantin – was substantially enriched with the triple mutant c-Myc–TRC40_D74E/L190D/I193D_ (Fig. 4). This behavior may suggest an interaction with TRC40 that does not depend on the TA-binding groove. By using an antibody against endogenous giantin that worked well in indirect immunostaining but not western blotting, we detected the protein in HeLa cells expressing wild-type, single, or the triple mutant forms of TRC40 (Fig. 5A). We observed a diffuse and reduced Golgi staining pattern when either the TRC40_D74E_ or the triple mutant were expressed. Hence, giantin behaved similarly to Stx5 but, unlike it, did not react to the manipulation of the TA-binding groove. We hypothesized that the unusual presence of a histidine in the TMS of giantin (Fig. 5B) could explain the effect of the triple mutant on the subcellular localization of the protein. The exchange of two hydrophobic residues to aspartate residues creates a negative patch in the center of the TA-binding groove. Inspection of the TMS of other TA golgins, that is golgin-84 and CASP (Gillingham and Munro, 2016), revealed a histidine residue in the same position (Fig. 5B). We tested whether these proteins interacted with TRC40_D74E_ or the triple mutant by performing an immunoprecipitation assay (Fig. 5C,D). Indeed, golgin-84 and CASP interacted with both TRC40 variants or even preferred the triple mutant in the case of the golgin CASP.

To test whether this preference involved a charge interaction between the histidine and the negatively charged TMS-binding groove, we introduced positively charged side chains and created the triple mutant c-Myc–TRC40_D74E/L190K/I193K_, which should make binding of an TMS containing a histidine energetically unfavorable. In the case of golgin-84 but not CASP, this reduced the interaction substantially with respect to co-purification with TRC40_D74E_ or the negatively charged triple mutant (Fig. 5C,D). With respect to golgin-84, our result confirms a report by Casson et al. (2017) who also described an interaction of the protein with TRC40 (without testing its dependence TMS-binding groove) but observed no effect on its steady-state localization in a TRC40-knockout cell line (Casson et al., 2017). In summary, we conclude from our data that the TA golgins fall into the class of substrates that can be targeted by the TRC pathway but do not absolutely require it for correct subcellular localization (Casson et al., 2017). Interestingly, we observed strong reductions in the steady-state levels of both CASP and golgin-84 upon combined silencing of *WRB* and *TRC40* (Fig. S7), further corroborating the notion that the TRC pathway is highly relevant to their turnover. While our results plausibly support a model where TRC40 can target the TA protein golgins, they also highlight that a proportion of the observed TRC40-co-purifying pool may reflect an interaction in a functional context distinct from precursor targeting. Given the important roles of Stx5, Stx6 and the golgins in Golgi morphology and function more work will be required to determine how direct and indirect effects combine to result in the observed reduction at the steady-state.

**Fig. 6.**
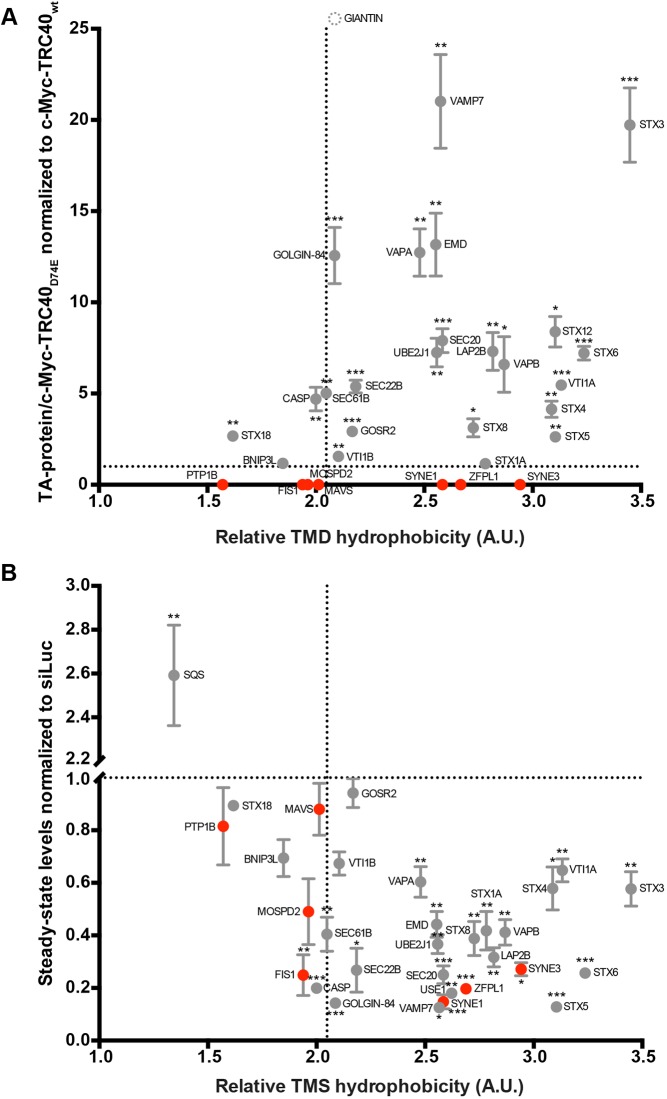
**TMS hydrophobicity determines but does not fully explain TRC40 dependence.** (A) Summary of the trapping efficiency observed for different TA proteins with c-Myc–TRC40_D74E_ as compared to wild-type c-Myc–TRC40 (*n*≥3, mean*±*s.e.m. shown) plotted against the hydrophobicity score of the TMS (GRAVY scale; Kyte and Doolittle, 1982). (B) Effects of combined siRNA-mediated downregulation of *TRC40* and *WRB* on the depicted TA proteins (*n*≥3, mean±s.e.m. shown) according to their relative TMS hydrophobicity score determined through the GRAVY scale. *P<0.05; **P<0.01; ***P<0.001 (two-tailed *t*-test). Giantin is depicted with a dashed symbol on top of the scheme to indicate that its TMS hydrophobicity score is similar to the other two TA golgins. The dotted line (*x* axes) indicates the hydrophobicity score of Sec61β TMS and (*y* axes) the same ratio of TA protein normalized to the pertinent control. TA proteins that were not trapped by TRC40_D74E_ are depicted in red.

Fig. 6 summarizes the data for the whole TA protein panel that we investigated in experiments probing the enrichment in the TRC40_D74E_ mutant immunoprecipitation (Fig. 6A based on data in Figs 3 and 4, Figs S4 and S5 and Table 1) or TA protein steady-state levels upon combined *TRC40* and *WRB* silencing (Fig. 6B; based on data in Fig. 1 and Fig. S7) plotted against the hydrophobicity of the respective TMS. A dotted line indicates the hydrophobicity score of the Sec61β TMS, previously proposed as the dividing line that determines TRC40-dependence of a TA protein precursor (Guna et al., 2018). We chose the GRAVY score (Kyte and Doolittle, 1982) instead of the transmembrane tendency score (Zhao and London, 2006) to describe the properties of the TMSs. The main difference between the two scores lies in the weight allocated to aromatic residues, which frequently occur in membrane proteins but are less hydrophobic than aliphatic side chains. However, no systematic differences were observed when correlating our experimental results with the series of TMS scores determined by one or the other method (data not shown).

While both experimental parameters generally correlate well with TMS hydrophobicity, that is most TA proteins with a TMS more hydrophobic than Sec61β do interact with TRC40 and respond to the manipulation of the TRC pathway, the dividing line defines a region of TMS hydrophobicity where it is impossible to predict whether a TA protein is affected by the trap or the silencing. In fact, our data provide examples of proteins that interacted but whose steady-state levels were not affected (Stx18 and GOSR2) or vice versa (Fis1 and MOSPD2). Furthermore, we found TA proteins with TMSs in a hydrophobicity range identical to *bona fide* substrates (SYNE1, SYNE3 and ZFPL1; red dots in Fig. 6A based on Fig. S4C) that we were unable to trap using TRC40_D74E_ although their steady-state levels were strongly affected when the TRC pathway was not operational. In conclusion and after careful consideration of the caveats associated with either experimental strategy, the data presented in Fig. 6 allow a reassessment of the properties of a TA protein relevant to its interaction with TRC40, which may represent a combination of interactions during biogenesis, including membrane targeting and other functional contexts.

## DISCUSSION

*In vitro* approaches have been extensively used to study the functional interaction of TRC40 with different TA proteins (Favaloro et al., 2008, 2010; Johnson et al., 2012; Leznicki et al., 2010, 2011; Mariappan et al., 2011; Rabu et al., 2008; Stefanovic and Hegde, 2007). The hydrophobicity of the TMS has been considered a key factor for organelle targeting (Borgese et al., 2001, 2003, 2007; Bulbarelli et al., 2002) as well as for discriminating between different targeting pathways to the same subcellular compartment (Costello et al., 2017b; Guna and Hegde, 2018; Guna et al., 2018; Rabu et al., 2008, 2009; Yagita et al., 2013). The diversity of the targeting pathways raises the question of which pathway is in charge of the biogenesis of a given TA protein when all pathways are operational.

In order to address this problem, we developed an ATPase-impaired dominant-negative mutant of TRC40 that is able to trap TA proteins in the cytoplasm. This means, for the first time, it has been possible to perform co-immunoprecipitation of TRC40 to identify the interactions with endogenous TA proteins without the use of crosslinkers. This tool enables us to identify the *in vivo* TA protein interactome of TRC40 in human cells (Fig. 6A). We used both candidate testing as well as unbiased identification of the most enriched targeting clients. We identified novel TRC40 targets, such as VAPA and VAPB, which are involved in membrane contact sites and lipid exchange between organelles (Costello et al., 2017a; Dong et al., 2016; Hua et al., 2017; Manford et al., 2012; Rocha et al., 2009), and the subclass of coiled-coil forming golgins, such as giantin, golgin-84 and CASP, that are tethered to the medial Golgi through a C-terminal TMS. Although the interpretation of the TRC40 interaction that we observed in terms of biogenesis is consistent with all experimental evidence and the current model of TRC40-dependent targeting, we cannot exclude that the trapped intermediates may also reflect an intermediate of another TRC40-related pathway, that is, in the context of cellular quality control or proteostasis. In fact, the interaction of TRC40_D74E_ with the TA protein golgin CASP did not depend on an intact hydrophobic groove thought to accommodate the TMS of TA proteins (Fig. 5C,D) suggesting that TRC40 may recognize another aspect of this golgin.

In line with the proposed cut-off for TRC40 interaction (Guna et al., 2018), we have trapped one TA protein with a TMS hydrophobicity score substantially lower than that of Sec61β, namely, Stx18 (1.62 versus 2.05) based on the GRAVY score to determine TMS hydrophobicity (Kyte and Doolittle, 1982). However, the transmembrane tendency scores (Zhao and London, 2006) for the TMS of Sec61β and Stx18 are similar (21.45 versus 21.62). Generally, this does not mean that transmembrane tendency is a better predictor of the ability of a TA protein to interact with TRC40 since the UBE2J1 and GOSR2 TMS score substantially lower than Sec61β using transmembrane tendency but were nevertheless trapped (this was better predicted by the GRAVY score where they show a higher hydrophobicity than Sec61β, Fig. 6A). As expected based on the hydrophobicity of the TMS by either score, we exclude PTP1B as a TRC40 client; only marginal effects on its steady-state levels were observed upon combined *WRB* and *TRC40* silencing and it did not co-immunoprecipitate with the trapping mutant of TRC40, although it was detected readily in the cell lysates. This is consistent with the finding that PTP1B is able to spontaneously insert into liposomes (Brambillasca et al., 2006). The two mitochondrial proteins investigated, BNIP3L and Fis1, were not enriched with the trap mutant (Fig. S4B,C). However, their steady-state levels reacted differently to the combined silencing of *TRC40* and *WRB*; this manipulation strongly reduced the steady-state levels of Fis1 but not BNIP3L (Fig. S7). Taken together, these results are consistent with the notion that mitochondrial TA proteins do not use the TRC pathway (Figueiredo Costa et al., 2018). Hence, the effect on Fis1 steady-state levels may relate to another function of TRC40.

We identified three TA proteins, SYNE1, SYNE3 and ZFPL1, that were not trapped by TRC40_D74E_ (Fig. S4C, Fig. 6A) despite a TMS hydrophobicity score that would have predicted an interaction with TRC40 during biogenesis. We cannot exclude that such a failure to be trapped may reflect a low amount of TA protein precursor present in the cell, although ZFPL1 has been shown to readily detected in HeLa and HEK293 cells (Cho et al., 2018; Geiger et al., 2012). Generally, we found no indication that copy numbers or half-life – as available from systematic studies on protein turnover in HeLa cells (Cambridge et al., 2011; Cho et al., 2018) – correlated with the propensity of a TA protein to be trapped by TRC40_D74E_. For example, TA proteins identified by SWATH mass spectrometry with high confidence ranged from 140 million molecules per cell and a half-life of 47 h (EMD) to 3 million and 20 h (Stx5) or even only 800,000 molecules per cell (UBE2J1). In fact, based on the available data (Cambridge et al., 2011; Cho et al., 2018) VAPB (80 million molecules per cell and 42 h) and FIS1 (56 million and 40 h), and GOSR2 (10 million, 29 h) and PTP1B (6 million, 31 h) are pairs of TA proteins of very similar abundance and half-life that perfectly underscore TMS hydrophobicity as the dominant parameter as a prediction for TRC40 recruitment in live cells.

The TRC40 triple mutant we generated disturbs the hydrophobic pocket of TRC40. Consistent with the expectation that this manipulation would reduce the affinity of TRC40_D74E_ for TA proteins we observed a strong reduction in co-immunoprecipitation of most TA protein substrates (Figs 3D and 4, Figs S4 and S5). However, the two TA golgins golgin-84 and CASP were clear exceptions as they co-purified equally or even more strongly with the triple mutant as compared to TRC40_D74E_. We attribute this to a central histidine within the TMS of the TA golgins that may be able to interact electrostatically with the mutant, now negatively charged, binding pocket of TRC40. Consistent with this notion, golgin-84 interacted substantially less with a charged-inverted triple mutant TRC40_D74E/L190K/I193K_. In contrast, CASP co-purified more extensively with either triple mutant compared to TRC40_D74E_. This suggests that the interaction between TRC40 and CASP is independent of the TA-binding groove and may instead be based on the chaperone activity of TRC40.

The yeast GET pathway proteins are encoded by non-essential genes (Schuldiner et al., 2008) despite the fact that a strongly Get3-dependent substrate, namely Sed5 (yeast Stx5), is encoded by an essential gene (Giaever et al., 2002). The phenotypes of *get* deletion mutants become apparent under stress conditions such as heat or oxidative stress (Kiktev et al., 2012; Metz et al., 2006). In contrast, the TRC pathway is essential in mammalian cells (Mukhopadhyay et al., 2006; Tran et al., 2003). The identification of novel TA protein substrates presented here provides new avenues into understanding why this system is so important in multicellular organisms; VAPA, encoded by a gene essential for early embryonic development in the mouse (McCune et al., 2017), and VAPB are important for membrane contacts and lipid homeostasis (Costello et al., 2017a; De Vos et al., 2012; Hua et al., 2017). The TA protein subgroup of golgins plays a major role in organizing vesicular traffic between the different cisternae of the Golgi (reviewed in Munro, 2011). They are key determinants of the post-translational modification of secretory proteins in the Golgi. In particular, giantin is required for the proper localization and activity of Golgi-resident glycosyltransferases (Stevenson et al., 2017). Together with SNARE proteins – active in the Golgi and the endosomal pathway – the client spectrum of the TRC pathway suggests a scope that will not only affect the trafficking, but also the post-translational modification of secretory proteins, and the homeostasis of the endomembrane system at large.

**Fig. 7.**
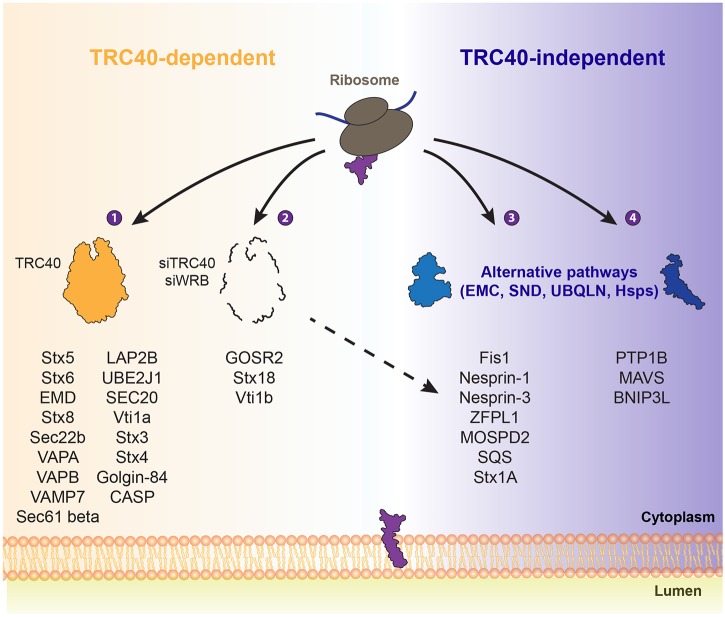
**TRC40 dependence of TA protein targeting.** Based on the interaction with TRC40_D74E_ and the steady-state levels when the TRC pathway is impaired (Figs 1, 4, 5 and 6; Figs S2, S3, S4, S6 and S7), TA proteins can be categorized in the following groups. (1) Substrates that can interact with TRC40_D74E_ and whose levels are affected at the steady-state when the pathway is downregulated. Therefore, TRC40 is likely involved in the targeting of those proteins. (2) TA proteins that can interact with TRC40_D74E_ but that remain unaltered upon the knockdown of the pathway. Even though they can interact with TRC40, there is redundancy and robust targeting in the absence of the TRC pathway. (3) Despite of the lack of interaction with TRC40, the combined knockdown of *WRB* and *TRC40* affects the steady-state levels of this group of TA proteins. Hence, the decrease of their protein steady-state levels most likely reflects an indirect effect. (4) Other pathways target these TA proteins that are unable to interact with TRC40 and their steady-state levels are unaffected when the TRC pathway is impaired.

Our findings offer a satisfying synthesis of information on TA protein targeting obtained by different approaches (Fig. 7). Even though it shows redundancy with other targeting options, the TRC pathway represents a robust route to the ER and accommodates a large client spectrum (Fig. 7, group 1). The precise flux may differ from cell type to cell type: while Stx8 steady-state levels were not affected in a *Wrb*-knockout mouse model (Rivera-Monroy et al., 2016) the protein was reduced in HeLa cells upon silencing of *TRC40* and *WRB* (Figs 1 and 6B). For some clients the TRC pathway may be nothing but a back-up option (group 2). In several cases TRC pathway manipulation affected the steady-state levels of proteins (Fig. 7, group 3) but no interaction with the TRC40 trap was observed. The explanation may differ for the different TA proteins in this group, ranging from indirect effects to a very low rate of biogenesis. Some of the TA proteins in this group feature a TMS hydrophobicity score that is easily compatible with TRC40-dependent targeting (the nesprins SYNE1 and SYNE3 and ZFPL1) and it will be interesting to obtain further insights into their biogenesis.

An important dimension that is rarely studied in the context of targeting experiments is the functional outcome of targeting by different pathways for individual proteins. After biogenesis and to fulfill their cellular functions, the golgins, Stx5 and the VAPs all undergo many diverse and often reversible protein–protein interactions resulting in coiled-coils, SNARE bundles or dynamic tethers between two membranes. Since a chaperone holdase activity of Get3 has been reported (Voth et al., 2014), it may be worth considering that the TRC pathway could aid its clients beyond targeting by chaperoning their cytoplasmic domains. In the case of Stx5, we have provided some evidence that critical determinants of TRC40-dependence may map to the N-terminal domain of the TA protein (Rivera-Monroy et al., 2016). So far, all analyses demonstrating correct targeting of a TA protein in the absence of the TRC pathway conclude that targeting was covered by another pathway, because no effect on the steady-state levels or localization of the protein was observed. However, the functionality of TA proteins that can be targeted by TRC40 but do not strictly require it has never been explored, namely with respect to the protein–protein interactions of their N-terminal domains or their dynamic behavior within the membrane. We propose that a full understanding of TA protein targeting will eventually include mechanisms that cater to the function of specific TA proteins in addition to ensuring that they are brought to the right membrane during the initial phase of biogenesis.

## MATERIALS AND METHODS

### Plasmids and constructs

All constructs were obtained using standard molecular biology methods and all sequences were verified by Sanger sequencing (GATC Biotech, Konstanz, Baden-Württemberg, Germany).

pcDNA3.1(-)_c-Myc-TRC40 was obtained after subcloning it from MBP-TEV-TRC40/ZZ-EMD-opsin, previously described (Favaloro et al., 2010), into a pcDNA3.1(-) (Invitrogen, Carlsbad, CA) vector by using overlap extension PCR. Primers for mutagenesis of TRC40 contained a sequence for making it insensitive to the siRNA (Catalog#: s1675; Ambion, Austin, TX) that was described previously (Pfaff et al., 2016; Rivera-Monroy et al., 2016). First, one PCR was performed using a forward primer containing an N-terminal c-Myc-tag for TRC40 and a restriction site for XhoI (5′-ATACTACTCGAGATGGAGCAGAAACTCATCTCTGAAGAGGATCTGATGGCGGCAGGGGTGGCC-3′) and the reverse primer (5′-TTGGGAAATAAAGGGGGAGATCTGGTTCTTGATCTGCAT-3′), which contained the sequence to be mutagenized. Second, another PCR using a forward primer (5′-TCCCCCTTTATTTCCCAAATGTGCAACATGCTGGGCCTGG-3′), overlapping the sequence of the aforementioned reverse, and a reverse primer targeting the C-terminus of TRC40 and containing a restriction site for BamHI (5′-ATACTAGGATCCCTACTGGGCACTGGGGGGCT-3′) was performed. A third PCR using the products of the first two PCRs was the performed. The PCR product was cloned into pcDNA3.1(-) using BamHI and XhoI.

The pcDNA3.1(-)_c-Myc-TRC40 mutants were obtained by site-directed mutagenesis using the following primers: D74E forward primer (5′-GTGTTCTGATCATCTCCACAGAGCCAGCACACAACATCTCAG-3′) and reverse primer (5′-CTGAGATGTTGTGTGCTGGCTCTGTGGAGATGATCAGAACAC-3′); D74N forward primer (5′-GTGTTCTGATCATCTCCACAAACCCAGCACACAACATCTCAG-3′) and reverse primer (5′-CTGAGATGTTGTGTGCTGGGTTTGTGGAGATGATCAGAACAC-3′); I193D forward primer (5′-CCTGGGCCGGCTTATGCAGGACAAGAACCAGATCTCC-3′) and reverse primer (5′-GGAGATCTGGTTCTTGTCCTGCATAAGCCGGCCCAGG-3′); L190D/I193D forward primer (5′-GGAGCGGGGCCTGGGCCGGGACATGCAGGACAAGAACCAG-3′) and reverse primer (5′-CTGGTTCTTGTCCTGCATGTCCCGGCCCAGGCCCCGCTCC-3′); L190K/I193K forward primer (5′-CGTGGAGCGGGGCCTGGGCCGGAAGATGCAGAAGAAGAACCAGATCTCCCCCTTTATTTCC-3′) and reverse primer (5′-GGAAATAAAGGGGGAGATCTGGTTCTTCTTCTGCATCTTCCGGCCCAGGCCCCGCTCCACG-3′).

The plasmid pGEM3Z-Stx5-opsin for the *in vitro* transcription and translation assays was as described previously (Rivera-Monroy et al., 2016). For pET23b_c-Myc-TRC40 constructs pcDNA3.1(-)_c-Myc-TRC40 plasmids were digested with NheI and HindIII and the inserts were ligated into pET23b. For pcDNA5/FRT/TO_Stx5-opsin construct, pGEM3Z-Stx5-opsin was digested with KpnI and XhoI, and the inserts were ligated into a pcDNA5/FRT/TO vector.

### Cell culture

HeLa P4 cells (Charneau et al., 1994) were obtained from the NIH AIDS Reagent Program. HeLa P4 and T-REx 293 Stx5-op cells were grown in DMEM (Gibco, Carlsbad, CA) supplemented with 2 mM L-glutamine (Gibco) and 10% (v/v) FBS (Biochrom, Berlin, Germany) (DMEM++) in a 37°C incubator at 5% CO_2_. They were tested for contamination by *Mycoplasma* spp. on a regular basis.

### T-REx 293 Stx5-opsin cell line generation

Flp-In T-REx 293 cells (Invitrogen) were cultured with DMEM supplemented with 10 μg/ml blasticidin. pOG44 and pcDNA5/FRT/TO_Stx5-opsin plasmids were co-transfected. At 2 days after transfection, cells were selected by incubating with DMEM++ supplemented with 15 μg/ml blasticidin and 200 μg/ml hygromycin B for 2 weeks until obtaining positive clones. The expression of Stx5–op from the stable transfectants was tested by western blotting. For inducing the expression of Stx5–op, 1 μg/ml tetracycline was added into the medium and the cells were incubated at 37°C for 6 h.

### Cell transfection and knockdown

HeLa P4 or T-REx 293 cells were seeded so as to reach 60–80% confluence on the day of transfection. Lipofectamine 2000 (Invitrogen) was used for transfections. Cell were incubated for 6 h and then harvested 48 h after Lipofectamine 2000 transfection.

Small interference RNA (siRNA) was used for the knockdown of *TRC40* and *WRB*. Specific RNA oligonucleotides against WRB and TRC40 were described previously (Rivera-Monroy et al., 2016) and luciferase siRNA (sense: 5′-CGUACGCGGAAUACUUCGA-3′, antisense: 5′-UCGAAGUAUUCCGCGUACG-3′, Sigma-Aldrich) was used as control. For the knockdown, cells were transfected twice at 48 h interval using Lipofectamine RNAiMAX (Invitrogen). Cells were incubated for 24 h with the RNAiMAX-siRNA solution and harvested 48 h after the final transfection.

### Cell fractionation

The lysis of the cells was performed with a dounce homogenizer and the lysate was centrifuged for 1 h at 180,000 ***g*** at 4°C for sedimenting the membranes. The pellet was resuspended and centrifuged again at 180,000 ***g*** for 20 min at 4°C. The cytosolic and membrane fractions underwent protein extraction with solubilization buffer, as previously described (Kline et al., 2009). Finally, cytosolic and membrane fractions were subjected to TCA precipitation, and proteins later resuspended in SDS loading buffer.

### Indirect immunofluorescence

Cells were grown in coverslips, fixed with 4% (w/v) paraformaldehyde (PFA) in PBS for 15 min and then permeabilized with 0.05% SDS in PBS containing 0.3% Triton X-100 for 10 min at room temperature. Cells were blocked in 10% FBS in PBS for 30 min and incubated with primary antibodies diluted in 5% FBS in PBS overnight at 4°C. Incubation with Alexa Fluor secondary antibodies (Invitrogen) was performed for 60 min at room temperature. The samples were mounted with Mowiol-DAPI. The list of antibodies used can be found in Table S1. Cells were analyzed using an Axiovert 200 M fluorescence microscope with a 63× Plan-Neofluar 1.3 NA water-corrected objective and appropriate filter settings. Images were acquired by using a LSM 510-META confocal laser scanning microscope (Zeiss, Jena, Thuringia, Germany). For confocal imaging a UV laser (405 nm) at 25 mW, a tunable argon laser (488 nm) at 30 mW, HeNe laser line (543 nm) at 1 mW, HeNe laser lines (633 nm) at 3 mW were used for excitation. Emission filters: 450/60 nm, 518/25 nm, 588/56 nm, long-pass (LP) 650 nm, respectively. The analysis of the images was performed with ImageJ 1.51w software (US National Institutes of Health; Schneider et al., 2012).

### Digitonin semi-permeabilization

HeLa P4 cells on coverslips were semi-permeabilized with a solution of 0.007% of digitonin in transport buffer [20 mM HEPES, 110 mM KOAc, 2 mM Mg(OAc)_2_, 1 mM EGTA, pH 7.3, 2 mM DTT, 0.1 mM PMSF and 1 µg/ml each of leupeptin, pepstatin and aprotinin] for 5 min on ice in living cells prior to fixation. Control cells were permeabilized with 0.3% Triton X-100 and 0.05% SDS in PBS after PFA fixation.

### TRC40 co-immunoprecipitation

Cells expressing empty vector (EV), wt, D74E, D74E/L190D/I193D, D74E/L190K/I193K c-Myc-tagged TRC40 variants from 15 cm dishes were homogenized in a Polytron homogenizer in 500 μl IP buffer (50 mM HEPES, 150 mM NaCl, 1.5 mM MgCl_2_, 1 mM EGTA, pH 7.4); 2.5 mg of total lysate was diluted with IP buffer to 1.2 ml and centrifuged at 180,000 ***g*** for 1 h to obtain the cytosolic fraction.

200 μl of cytosol was used as input. The rest, ∼1 ml, was incubated for 2.5 h in a rotation wheel with 50 μl Pierce anti-c-Myc agarose resin (Thermo Fisher Scientific) previously blocked with 5% BSA in IP buffer for 1 h. The resin was transferred to 600 μl columns and washed three times (2 min each) prior to elution with 50 μl 1× loading buffer plus 100 mM DTT.

### Mass spectrometric analysis

c-Myc–TRC40 immunoprecipitates from HEK293T cells transfected with c-Myc–TRC40 constructs were reconstituted in 1× NuPAGE LDS Sample Buffer (Invitrogen) and applied to 4–12% NuPAGE Novex Bis-Tris Minigels (Invitrogen). For quantification, samples were run 1 cm into the gel for purification. For generation of a spectral library, pooled aliquots of biological replicates for each sample were run half-distance through the gel. Gels were stained with Coomassie Blue for visualization purposes, and the library lanes cut into 12 equidistant slices regardless of staining. Quantification samples were processed as a single slice. After washing, gel slices were reduced with dithiothreitol (DTT), alkylated with 2-iodoacetamide and digested with trypsin overnight. The resulting peptide mixtures were then extracted, dried in a SpeedVac, reconstituted in 2% acetonitrile and 0.1% formic acid (v/v) and prepared for nanoliquid chromatography tandem mass spectrometry (nanoLC-MS/MS) as described previously (Atanassov and Urlaub, 2013). All samples were spiked with a synthetic peptide standard used for retention time alignment (iRT Standard, Biognosys, Schlieren, Switzerland).

Protein digests were analyzed on a nanoﬂow chromatography system (Eksigent nanoLC425) hyphenated to a hybrid triple quadrupole-TOF mass spectrometer (TripleTOF 5600+) equipped with a Nanospray III ion source (Ionspray Voltage 2400 V, Interface Heater Temperature 150°C, Sheath Gas Setting 12) and controlled by Analyst TF 1.7.1 software build 1163 (all Sciex, Framingham, MA). In brief, peptides were dissolved in loading buffer (2% acetonitrile and 0.1% formic acid in water) to a concentration of 0.3 µg/µl. For each analysis, 1.5 µg of digested protein were enriched on a precolumn (0.18 mm ID×20 mm, Symmetry C18, 5 µm; Waters, Milford, MA) and separated on an analytical RP-C18 column (0.075 mm ID×250 mm, HSS T3, 1.8 µm, Waters) using a 55 min linear gradient of 5–35% acetonitrile and 0.1% formic acid (v:v) at 300 nl min^−1^.

Qualitative LC-MS/MS analysis was performed using a Top25 data-dependent acquisition method with an MS survey scan of *m/z* 350–1250 accumulated for 350 ms at a resolution of 30,000 full-width at half maximum (FWHM). MS/MS scans of *m/z* 180–1600 were accumulated for 100 ms at a resolution of 17,500 FWHM and a precursor isolation width of 0.7 FWHM, resulting in a total cycle time of 2.9 s. Precursors above a threshold MS intensity of 125 cps with charge states 2+, 3+, and 4+ were selected for MS/MS, the dynamic exclusion time was set to 30 s. MS/MS activation was achieved by collision-induced ionization (CID) using nitrogen as a collision gas and the manufacturer's default rolling collision energy settings. One technical replicate per gel slice was analyzed to construct a spectral library.

For quantitative SWATH analysis, MS/MS data were acquired using 65 variable size windows (Zhang et al., 2015) across the 400-1050 *m/z* range. Fragments were produced using rolling collision energy settings for a charge state of 2+, and fragments acquired over an *m/z* range of 350–1400 for 40 ms per segment. Including a 100 ms survey scan, this resulted in an overall cycle time of 2.75 s. Three independent experiments each with three technical replicates were acquired for each biological state for quantitation purposes.

Protein identiﬁcation was achieved using ProteinPilot Software version 5.0 build 4769 (AB Sciex) at ‘thorough’ settings. A total of 541,419 MS/MS spectra from the combined qualitative analyses were searched against the UniProtKB human reference proteome (revision 04-2018, 93,609 entries) augmented with a set of 52 known common laboratory contaminants to identify 3135 proteins at a false discovery rate (FDR) of 1%.

Spectral library generation and SWATH peak extraction were achieved in PeakView Software version 2.1 build 11,041 (Sciex) using the SWATH quantification microApp version 2.0 build 2003. Following retention time correction using the iRT standard, peak areas were extracted using information from the MS/MS library at an FDR of 1% (Lambert et al., 2013). The resulting peak areas were then summed for each peptide and ﬁnally to the 2329 protein area values across all injections, which were used for further statistical analysis.

### Western blotting

Antibodies were diluted in blocking buffer (5% milk in PBS plus 0.1% Tween-20). The list of primary and secondary antibodies can be found in Table S1. The opsin monoclonal antibody (R2-15) was obtained from the laboratory of Bernhard Dobberstein from the University of Heidelberg and it was a kind gift from Paul A. Hargrave from the University of Florida, FL, and has been was described previously (Adamus et al., 1991). Anti-TRC40 #4 and -Sec61β antibodies were a kind gift from the laboratory of Bernhard Dobberstein (ZMBH, Heidelberg, Germany). Anti-BAG6 antibody was a kind gift from the laboratory of Stephen High (Faculty of Life Sciences, Manchester, UK). Blots were scanned using an Odyssey CLx infrared scanner (LI-COR Biosciences, Lincoln, NE) with IRDye LI-COR secondary antibodies (Table S1). The acquired images were analyzed and quantified with Image Studio 5.2.5 software (LI-COR).

### Coupled *in vitro* transcription and translation

Reactions were performed in the TnT Quick Coupled Transcription/Translation System (Promega, Madison, WI, USA) on TRC40-immunodepleted reticulocyte lysate (using rabbit anti-TRC40 #4) or rabbit IgG-treated lysate as control (Johnson et al., 2012; Leznicki et al., 2010; Pfaff et al., 2016). First, 150 ng of pET23b_c-Myc-TRC40 variants were added to 4.5 µl of each lysate and incubated for 30 min at 30°C (reaction #1). In the meantime, 250 ng of pGEM3Z_Stx5-op were added to a second set of 4.5 µl aliquoted lysates and kept on ice until use (reaction #2). Second, reactions #1 and #2 were combined and the transcription/translation was carried out for an additional 90 min at 30°C. Third, the ER integration assay was performed by adding 1 µl of pancreatic rough microsomes (RM) to the reaction mix and incubating it for 90 min at 30°C. Finally, the assay was stopped by adding 30 µl SDS sample buffer at 55°C and western blot analysis was performed for TRC40 and Stx5–op with 25% of the sample.

### Statistics

Data are presented as mean±s.e.m. Statistical analysis was assessed via an unpaired parametric two-tailed Student's *t*-test. To determine statistical significance (*P*<0.05) GraphPad Prism 6.0 (GraphPad Prism Inc., San Diego, CA) was used.

## Supplementary Material

Supplementary information
