# Supplementary material for: A trap mutant reveals the physiological client spectrum of TRC40
Source: J Cell Sci. 2019 Jul 1;132(13):jcs230094. doi: 10.1242/jcs.230094 (PMC6633398; doi:10.1242/jcs.230094)
Supplement: Supplementary information [file joces-132-230094-s1.pdf]

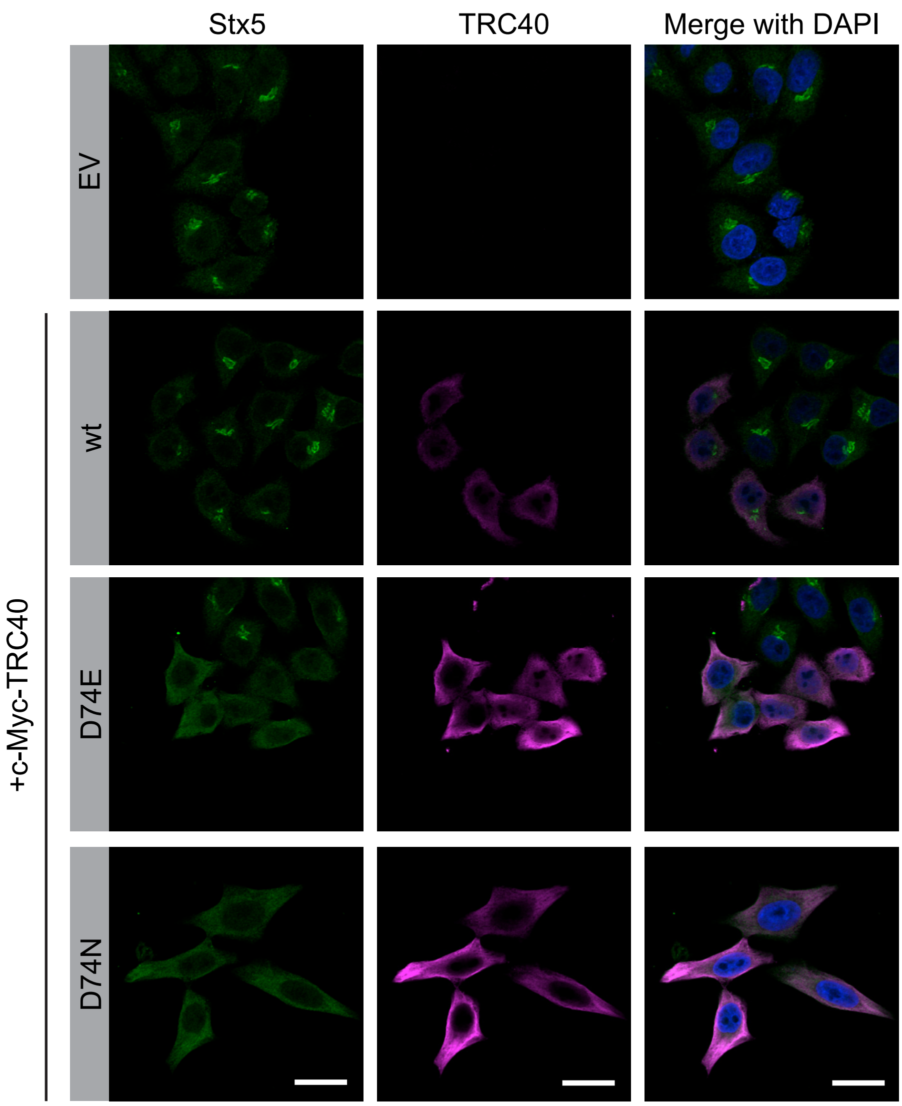

Figure S1. In the presence of TRC40$_{D74N}$ Stx5 accumulates in cytoplasm.
HeLa cells were transfected to express c-Myc-TRC40 constructs and later processed for indirect immunofluorescence using antibodies against TRC40 (magenta) and Stx5 (green). Transfection with the empty vector (EV) was used as negative control. The signal observed from endogenous TRC40 was close to background and it is therefore not visible with the settings used. Three independent biological replicates were analyzed. Scale bars: 20 μm.

Journal of Cell Science • Supplementary information

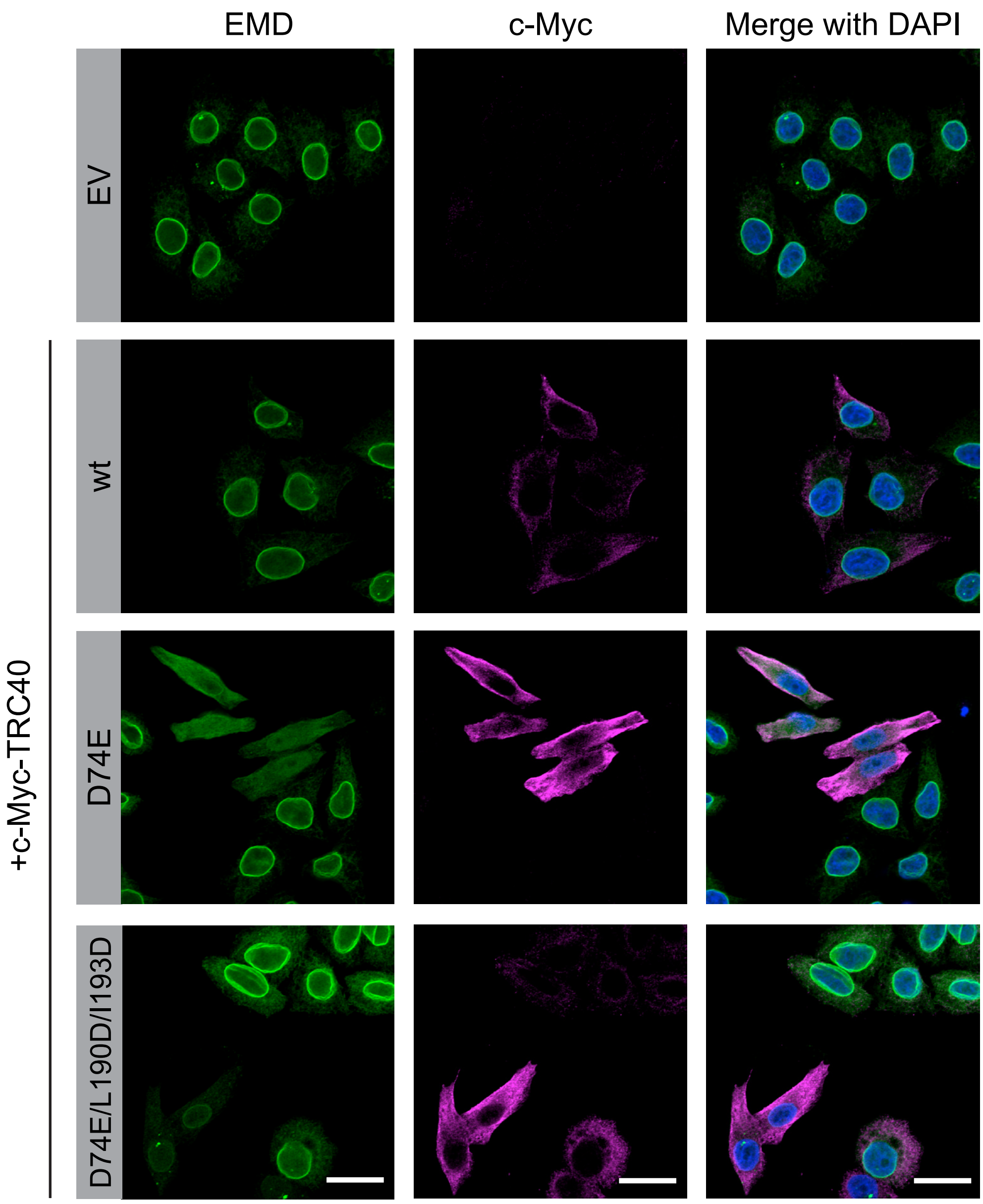

Figure S2. TRC40$_{D74E}$ traps EMD in the cytoplasm.
HeLa cells were transfected to express c-Myc-TRC40 constructs. Cells were subjected to indirect immunofluorescence using antibodies against c-Myc (magenta) and Stx5 (green). Three independent biological replicates were analyzed. Scale bars: 20 µm.

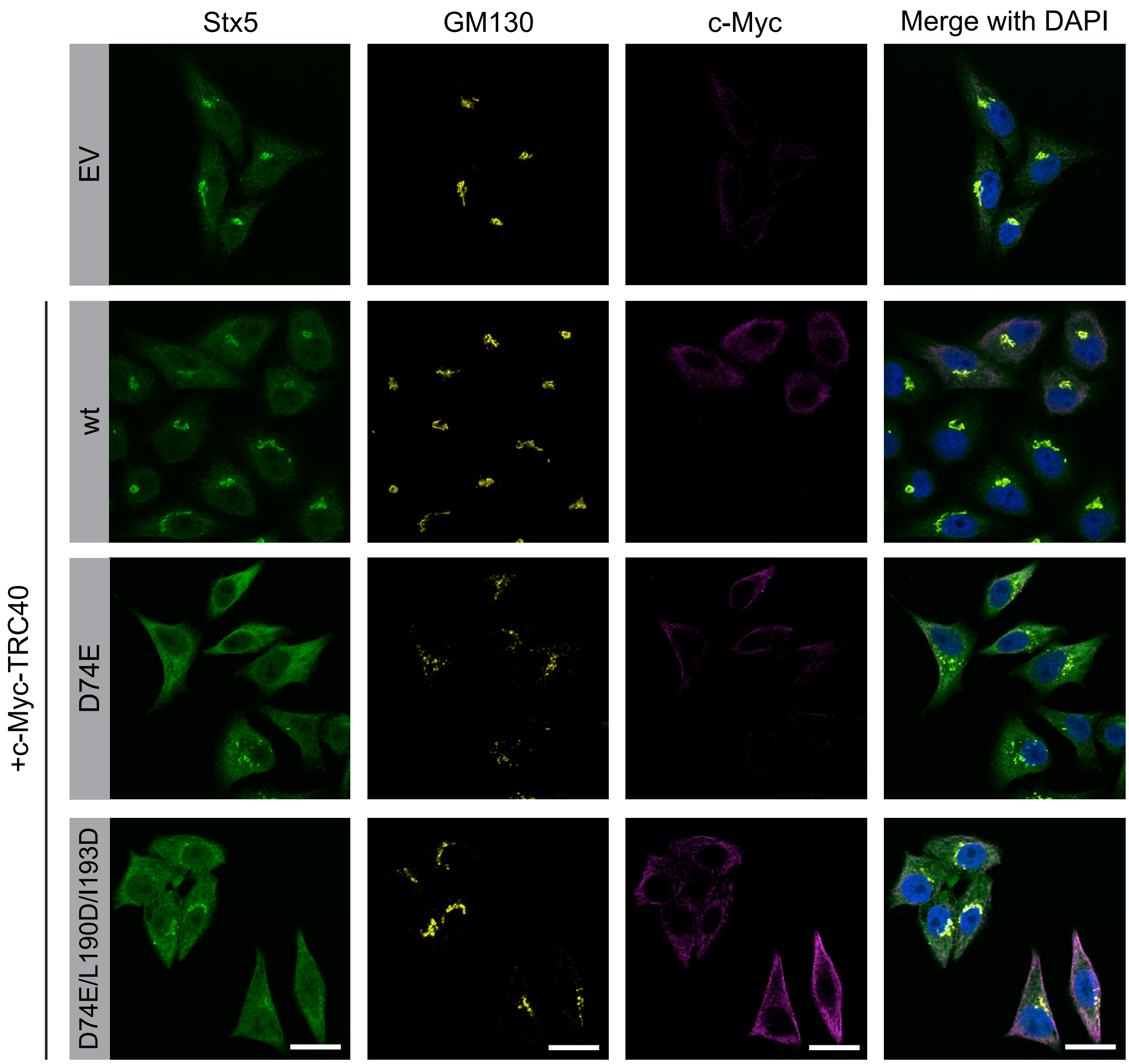

Figure S3. A double mutation in the TA-binding groove of TRC40$_{D74E}$ partially restores Stx5 localization.

HeLa cells were transfected to express c-Myc-TRC40 constructs. Cells were subjected to indirect immunofluorescence using antibodies against c-Myc (magenta), the *cis*-Golgi marker GM130 (yellow) and Stx5 (green). Three independent biological replicates were analyzed. Scale bars: 20 µm.

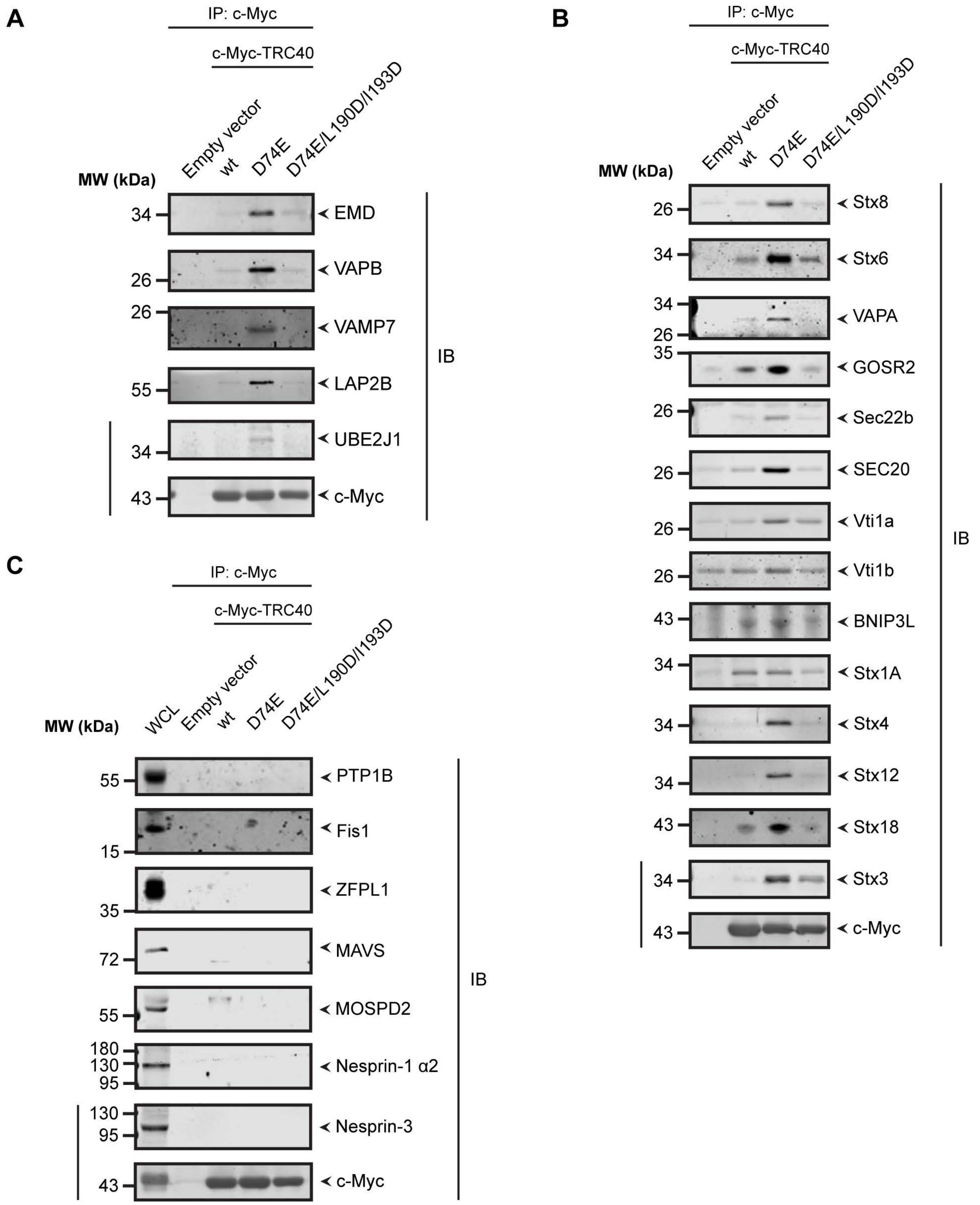

Figure S4. Candidate testing reveals many TA proteins interacting with the TRC40 trap mutant via the TA-binding groove.

(A-B) Co-immunoprecipitation from cytosol in the absence of detergent shows that $TRC40_{D74E}$ interacts with many TA-proteins. Cells were transfected with either c-Myc-TRC40, c-Myc-$TRC40_{D74E}$ or c-Myc-$TRC40_{D74E/L190D/I193D}$ using the empty vector (EV) as negative control. Western blot was performed detecting the indicated proteins. Three independent biological replicates were analyzed. The line indicates that the two panels are derived from the same lanes of the same gel. All other panels have a pertinent bait control used for the quantification but not shown here.(C) Detection of PTP1B, Fis1, ZFPL1, MAVS, MOSPD2, Nesprin-1 alpha 2 isoform and Nesprin-3 in the co-immunoprecipitated material reveals their absence from the c-Myc immuno-precipitates. Whole cell lysate (WCL) indicates the presence of the proteins in the transfected cells. The line indicates that the two panels are derived from the same lanes of the same gel. All other panels have a pertinent bait control used for the quantification but not shown here.

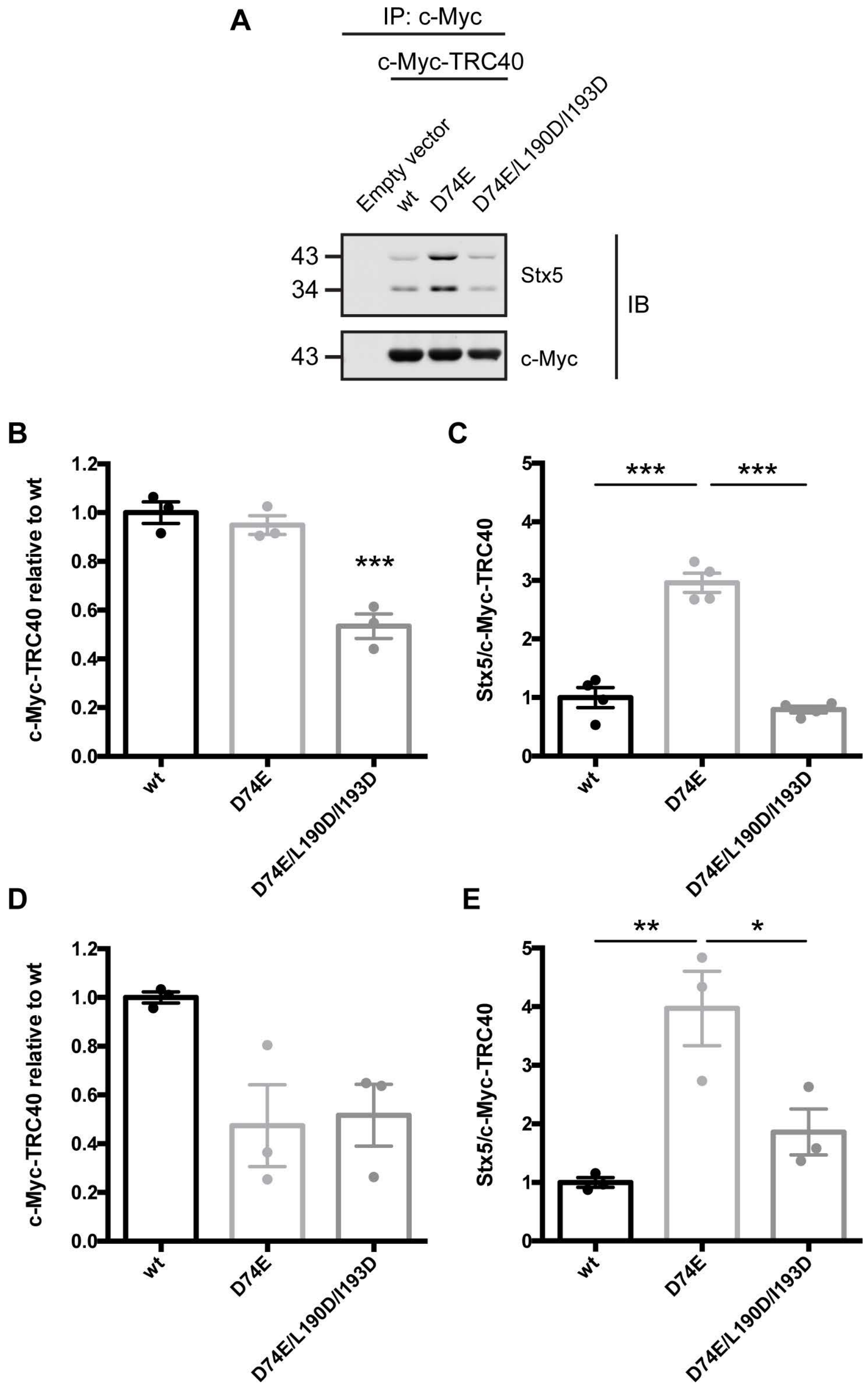

Figure S5. Quantitative SWATH mass spectrometry confirms TA-binding groove dependent enrichment of Stx5 with TRC40$_{D74E}$.
(A) Representative Western blots of eluates from anti-c-Myc-immunoprecipitation detecting the immunoprecipitated TRC40 variants and the co-precipitating TA protein Stx5. Note that endogenous Stx5 migrates as two different bands due to alternative start codons (Hui et al., 1997). At least three independent biological replicates were analyzed. (B) Quantification of c-Myc signal in immunoprecipitated material. The graph shows the mean and the error bars depict +-s.e.m.. Statistics were determined by two-tailed Student's *t*-test. Asterisks indicate statistical significance (*** $P < 0.001$). (C) Quantification of ratio between co-immunoprecipitated Stx5 and TRC40 variants. The graph shows the mean and the error bars depict +-s.e.m.. Statistics were determined by two-tailed Student's *t*-test. Asterisks indicate statistical significance (*** $P < 0.001$). (D) Peptide-based quantification of TRC40 variants from SWATH mass spectrometry analysis. Three independent biological replicates were analyzed. The graph shows the mean and the error bars depict +-s.e.m. (E) Quantification of ratio between co-immunoprecipitated Stx5 and TRC40 variants as obtained by SWATH mass spectrometry analysis. The graph shows the mean and the error bars depict +-s.e.m.. Statistics were determined by two-tailed Student's *t*-test. Asterisks indicate statistical significance (* $P < 0.05$; ** $P < 0.01$).

Journal of Cell Science • Supplementary information

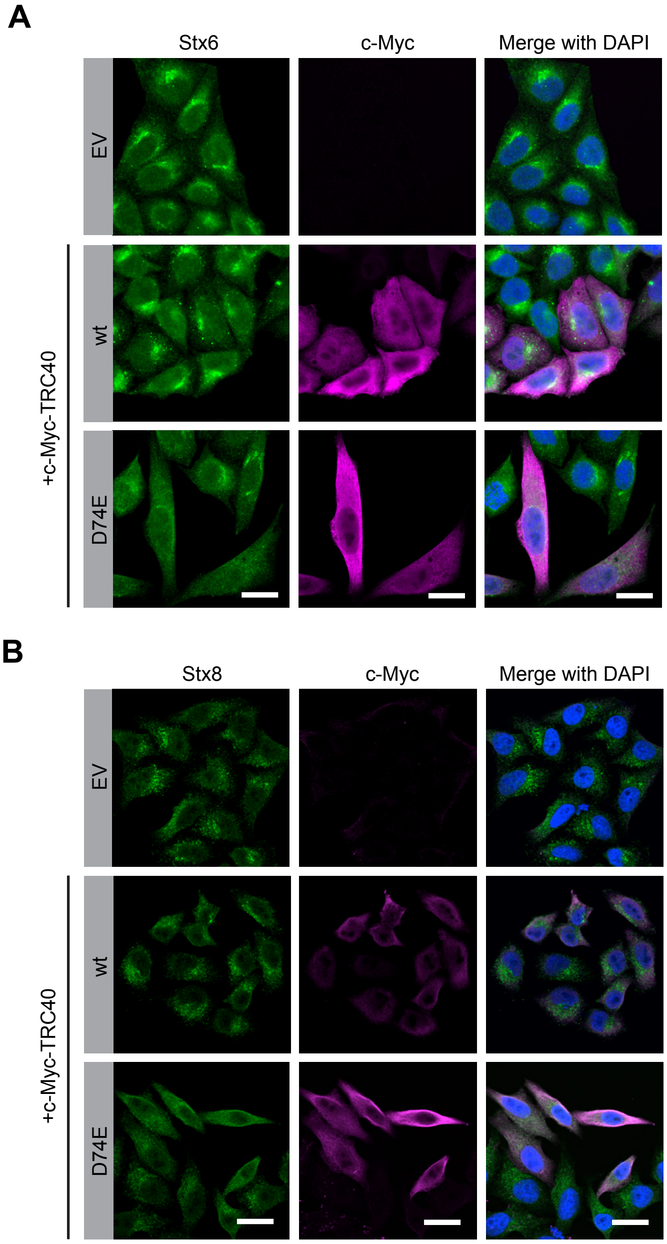

Figure S6. Stx6 and Stx8 are partially mislocalized upon over-expression of the TRC40 trap.

(A-B) HeLa cells were transfected to express c-Myc-TRC40 constructs and later processed for indirect immunofluorescence using antibodies against c-Myc (magenta), Stx6 (green, A) and Stx8 (green, B). Transfection with the empty vector (EV) was used as negative control. Three independent biological replicates were analyzed. Scale bars: 20 µm.

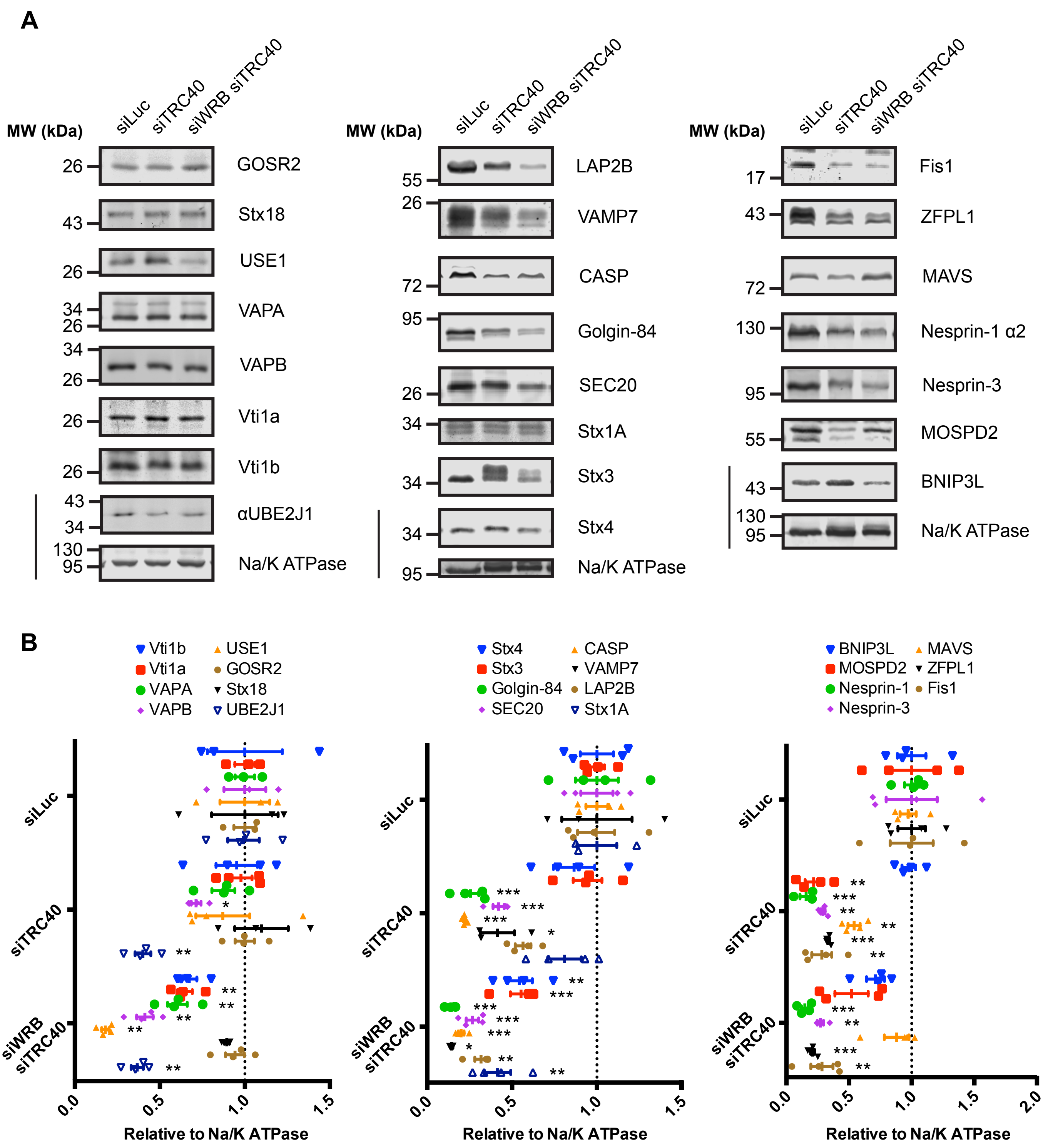

Figure S7. Silencing *WRB* and *TRC40* affects the steady-state levels of many TA proteins.
(A) Exemplary Western blots of the steady-state levels of different TA proteins after silencing *TRC40* or *TRC40* and *WRB*. The line indicates that the two panels are derived from the same lanes of the same gel. All other panels have a pertinent loading control used for the quantification but not shown here. (B) Quantifications of Western blots shown in A. All experiments were performed at least in biological triplicates. The plots depict the mean +- s.e.m.. Statistics were determined by two-tailed Student's *t*-test. Asterisks depict statistical significance (* $P < 0.05$; ** $P < 0.01$; *** $P < 0.001$). The dotted line indicates the siLuc steady-state levels.

Table S1. List of primary and secondary antibodies used in this study.

Name	Raised in	Company	Catalog no.	Lot #	Dilution (WB)	Dilution (IF)
BNIP3L	mouse	Invitrogen	39-3300	QC215886	1:250	
CAML	guinea pig	Synaptic Systems	359 004	359004/1	1:1000	
CASP	rabbit	Proteintech	11733-1-AP	50220	1:1000	
Emerin	rabbit	Santa Cruz	sc-15378	H1115	1:1000	1:150
Fis1	rabbit	Thermo Fisher	PA1-41082	QC1999106	1:1000	
GAPDH	mouse	NeoBiotech	NB-29-00852	16/06-G4-C5	1:40000	
Giantin	guinea pig	Synaptic Systems	263005	263005/1		1:200
GM130	mouse	BD Transduction Laboratories	610823	24277		1:300
Golgin-84	rabbit	Sigma-Aldrich	HPA000992	A104695	1:1000	
GOSR2	rabbit	Synaptic Systems	170003	170003/4	1:1000	
Lamin-A/C	mouse	abcam	ab40567			1:200
LAP2B	rabbit	Sigma-Aldrich	SAB2102484	QC14874	1:1500	
MAVS	mouse	Santa Cruz	sc-166583	L0417	1:500	
MOSPD2	rabbit	abcam	ab129250	GR89338-4	1:500	
c-Myc	mouse	Santa Cruz	sc-40	B0116	1:1000	1:200
c-Myc	rabbit	Santa Cruz	sc-789	I171		1:200
Na+/K+-ATPase α1	mouse	Santa Cruz	sc-21712	B1516	1:1000	
Nesprin-1	rabbit	abcam	ab192234	GR181158-5	1:1000	
Nesprin-3	rabbit	abcam	ab186751	GR163090-1	1:1000	
Opsin	mouse	From Bernhard Dobberstein			1:1000	
PTP1B	rabbit	Sigma-Aldrich	HPA012542		1:1000	1:100
SEC20	rabbit	abcam	ab151551	YJ081322CS	1:1000	
Sec22b	rabbit	Synaptic Systems	186003	186003/1-8	1:1000	
Sec61β	rabbit	From Bernhard Dobberstein			1:1000	1:300
Squalene synthase	mouse	Santa Cruz	sc-271602	D2816	1:1000	
Syntaxin 1	mouse	Synaptic Systems	110011	110011/13	1:1000	
Syntaxin 3	rabbit	Synaptic Systems	110033	110033/24	1:750	
Syntaxin 4	rabbit	Synaptic Systems	110041	110041/2	1:1000	
Syntaxin 5	rabbit	Synaptic Systems	110053	110053/16	1:2000	1:250
Syntaxin 6	rabbit	Synaptic Systems	110062	110062/9	1:1000	1:300
Syntaxin 8	rabbit	Synaptic Systems	110083	110083/1-11	1:1000	1:300
Syntaxin 12	rabbit	Synaptic Systems	110133	110133/2	1:1000	

Journal of Cell Science • Supplementary information

Syntaxin 18	rabbit	Synaptic Systems	110183	110183/3	1:1000	
TRC40	mouse	Sigma-Aldrich	WH0000439M3	D3011-2H3	1:1000	1:100
TRC40	rabbit	Proteintech	15450-1-AP	00021130	1:1000	
TRC40 #4	rabbit	From Bernhard Dobberstein			1:1000	
UBE2J1	mouse	Santa Cruz	sc-377002	H2917	1:1000	
USE1	rabbit	Proteintech	25218-1-AP	00022214	1:1000	
VAMP7	rabbit	Synaptic Systems	232003	232003/2-9	1:1000	
VAPA	rabbit	Proteintech	15275-1-AP	00022782	1:1000	
VAPB	rabbit	Proteintech	14477-1-AP	00013727	1:1000	
Vti1a	rabbit	Synaptic Systems	165003	165003/1	1:1000	
Vti1b	rabbit	Synaptic Systems	164002	164002/7	1:1000	
WRB	rabbit	Synaptic Systems	324002	324002/1-2	1:500	
ZFPL1	rabbit	Sigma-Aldrich	HPA014909	A117095	1:1000	

Short Name	Conjugated to	Company	Catalog no.	Lot #	Dilution
α-mouse HRP	HRP	Santa Cruz	sc-516102	F2017	1:10000
α-rabbit 800	IRDye 800CW	LI-COR	926-32213		1:5000
α-rabbit 680	IRDye 680LT	LI-COR	926-68023		1:5000
α-mouse 800	IRDye 800CW	LI-COR	926-32212		1:5000
α-mouse 680	IRDye 680LT	LI-COR	926-68050		1:5000
α-guinea pig 680	IRDye 680LT	LI-COR	926-32421		1:5000
α-guinea pig 488	Alexa Fluor 488	Invitrogen	A11073	1458631	1:1000
α-rabbit 488	Alexa Fluor Plus 488	Invitrogen	A32731	SE250296	1:1000
α-mouse 546	Alexa Fluor 546	Invitrogen	A11030	1829584	1:1000
α-mouse 647	Alexa Fluor 647	Invitrogen	A21235	1511346	1:1000
α-mouse 647	Alexa Fluor Plus 647	Invitrogen	A32728	SE250294	1:1000

Journal of Cell Science • Supplementary information

Table S2. TA-proteins pulled-down with the c-Myc-TRC40$_{D74E}$ construct analysed by mass-spectrometry.

UniProt ID	Protein name	Gene symbol	p value	log2 (Fold change)
**VAPB_HUMAN**	Vesicle-associated membrane protein-associated protein B/C	VAPB	0.0002	1.2995493
**EMD_HUMAN**	Emerin	EMD	0.0007	1.2408614
**VAMP7_HUMAN**	Vesicle-associated membrane protein 7	VAMP7	0.0008	2.3780926
**GOGB1_HUMAN**	Golgin subfamily B member 1 (Giantin)	GOLGB1	0.0035	-1.7250403
**LAP2B_HUMAN**	Lamina-associated polypeptide 2, isoforms beta/gamma	LAP2	0.0057	0.5583429
**K7ESP4_HUMAN**	Dephospho-CoA kinase domain-containing protein	DCAKD	0.0220	1.1499333
**UB2J1_HUMAN**	Ubiquitin-conjugating enzyme E2 J1	UBE2J1	0.0241	1.3633126
**STX5_HUMAN**	Syntaxin-5	STX5	0.0475	1.0932206
**CDKAL_HUMAN**	Threonylcarbamoyladenosine tRNA methylthiotransferase	CDKAL1	0.0753	0.8713599
**MAVS_HUMAN**	Mitochondrial antiviral-signaling protein (MAVS)	MAVS	0.2301	0.5362815
**INP4A_HUMAN**	Type I inositol 3,4-bisphosphate 4-phosphatase	INPP4A	0.5117	0.363725
**MCL1_HUMAN**	Induced myeloid leukemia cell differentiation protein Mcl-1	MCL1	0.5297	0.7643984
**E7EST9_HUMAN**	Ubiquitin carboxyl-terminal hydrolase 19	USP19	0.5301	0.6081325
**Q96NX8_HUMAN**	Syntaxin-16D	STX16	0.5515	0.408667
**LRC59_HUMAN**	Leucine-rich repeat-containing protein 59	LRRC59	0.6505	0.3805235
**FA98B_HUMAN**	Protein FAM98B	FAM98B	0.8603	0.180839
**FIS1_HUMAN**	Mitochondrial fission 1 protein	FIS1	0.8610	-0.0717239
**GOGA5_HUMAN**	Golgin subfamily A member 5 (Golgin-84)	GOLGA5	0.9002	0.0384847

Journal of Cell Science • Supplementary information

Table S3. Peptides obtained in MS and used for quantitation of statistically significant TA-proteins pulled-down with c-Myc-TRC40 constructs.

UniProt ID	Protein Name	Gene Symbol	Peptides used for Quantitation	Position	Unused Score	Total Score	Seq. Cov. [%]	PSMs > 95%
**O95292**	Vesicle-associated membrane protein-associated protein B/C	VAPB	SLSSSLDDTEVKK	156-68	17.72	17.74	51.8	11
			TVQSNSPISALAPTGK	201-216				
			VEQVLSLEPQHELK	4-17				
**P50402**	Emerin	EMD	DSAYQSITHYRPVSASR	158-174	12.68	12.72	25.2	9
			IFEYETQR	38-45				
			YNIPHGPVVGSTR	19-31				
**P51809**	Vesicle-associated membrane protein 7	VAMP7	AILFAVVAR	2-10	5.74	5.80	15.0	3
**Q9Y385**	Ubiquitin-conjugating enzyme E2 J1	UBE2J1	LSTSPDVIQGHQPR	265-278	8.43	8.45	21.1	4
**P42166**	Lamina-associated polypeptide 2, isoform alpha	LAP2	EATQILSVPK	526-535	77.05	77.21	65.0	55
			EPLVATNLPGR	254-264				
			ETTTGYYKDIVENICGR	316-332				
			GGTLFGGEVCK	675-685				
			QSQHDKIDASELSFPFHESILK	479-500				
			SGIQPLCPER	335-344				
			TVVSHSLTTLGLEVAK	463-478				
			VIEEEWQQVDR	501-511				
**P42167**	Lamina-associated polypeptide 2, isoforms beta/gamma	LAP2	GAAGRPLELSDFR	369-381	6.80	32.12	37.2	21
			GGPLQALTR	240-248				
			YVPLADVK	394-401				
**K7ESP4**	Dephospho-CoA kinase domain-containing protein	DCAKD	INAQLPLTDK	159-168	2.07	2.08	10.5	1
**Q13190**	Syntaxin-5	STX5	APVSALPLAPNHLGGGAVVLGAESHASK	214-241	26.08	26.16	45.1	17
			AVEIEELTYIIK	129-140				
			DLSNTFAK	104-111				
			DRTQEFLSACK	59-69				
			HLQTHSNTIVVSLQSK	167-182				
			NTDQGVYLGLSK	11-22				
			QIAQLQDFVR	149-158				
			YFQSVTSNR	325-333				
**Q14789**	Golgin subfamily B member 1	GOLGB1	AQVVDLLQQELTAAEQR	272-288	72.13	72.31	22.6	34
			GLTAQIQSFGR	2728-2738				
			SAAQPSTSPAEVQSLKK	2865-2881				

Unused Score: Sum of -log (confidence) values of all peptides per protein which is not claimed by other, non-overlapping hypotheses.
Total Score: Sum of -log (confidence) values of all peptides per protein.
PSMs >95%: number of primary sequences per protein substantiated by >95% confidence matches
